# Beyond the HLA polymorphism: a complex pattern of genetic susceptibility to pemphigus

**DOI:** 10.1590/1678-4685-GMB-2019-0369

**Published:** 2020-07-01

**Authors:** Maria Luiza Petzl-Erler

**Affiliations:** 1 Universidade Federal do Paraná, Departamento de Genética, Laboratório de Genética Molecular Humana, Curitiba, PR, Brazil.

**Keywords:** Pemphigus foliaceus, pemphigus vulgaris, blistering skin diseases, autoimmunity, genetic susceptibility

## Abstract

Pemphigus is a group of autoimmune bullous skin diseases that result in significant morbidity. As for other multifactorial autoimmune disorders, environmental factors may trigger the disease in genetically susceptible individuals. The goals of this review are to summarize the state of knowledge about the genetic variation that may affect the susceptibility and pathogenesis of pemphigus vulgaris and pemphigus foliaceus – both the endemic and the sporadic forms –, to compare and discuss the possible meaning of the associations reported, and to propose recommendations for new research initiatives. Understanding how genetic variants translate into pathogenic mechanisms and phenotypes remains a mystery for most of the polymorphisms that contribute to disease susceptibility. However, genetic studies provide a strong foundation for further developments in this field by generating testable hypotheses. Currently, results still have limited influence on disease prevention and prognosis, drug development, and clinical practice, although the perspectives for future applications for the benefit of patients are encouraging. Recommendations for the continued advancement of our understanding as to the impact of genetic variation on pemphigus include these partially overlapping goals: (1) Querying the functional effect of genetic variants on the regulation of gene expression through their impact on the nucleotide sequence of cis regulatory DNA elements such as promoters and enhancers, the splicing of RNA, the structure of regulatory RNAs and proteins, binding of these regulatory molecules to regulatory DNA elements, and alteration of epigenetic marks; (2) identifying key cell types and cell states that are implicated in pemphigus pathogenesis and explore their functional genomes; (3) integrating structural and functional genomics data; (4) performing disease-progression longitudinal studies to disclose the causal relationships between genetic and epigenetic variation and intermediate disease phenotypes; (5) understanding the influence of genetic and epigenetic variation in the response to treatment and the severity of the disease; (6) exploring gene-gene and genotype-environment interactions; (7) developing improved pemphigus-prone and non-prone animal models that are appropriate for research about the mechanisms that link genotypes to pemphigus. Achieving these goals will demand larger samples of patients and controls and multisite collaborations.

## Introduction

Pemphigus is a group of autoimmune skin diseases of unclear etiology, characterized by epidermal blisters and erosions in the stratified squamous epithelium affecting the skin and/or mucous membranes. The main forms are pemphigus vulgaris (PV) and pemphigus foliaceus (PF). Pemphigus patients produce immunoglobulin G (IgG) antibodies targeting proteins at the cell surface of keratinocytes. The autoantigens are part of the desmosomes, the molecular complexes specialized for cell-to-cell adhesion by anchoring intermediate filaments. Keratinocytes within pemphigus lesions lose cell-cell adhesion due to damage of desmosomes, a process named acantholysis. While PV can affect either the mucous membranes alone or the mucous membranes and the skin, in PF lesions develop only in the skin. The primary autoantigens are desmoglein 1 (DSG1) in PF and desmoglein 3 (DSG3) in PV, but PV patients may also develop anti-DSG1 autoantibodies. Detection of anti-desmoglein antibodies in patients with pemphigus is a hallmark and a diagnostic criterion. Additional autoantigens have been identified in PV patients (Kalantari-Dehaghi *et al.*, 2013; Sajda *et al.*, 2016); however, the significance of the non-desmoglein targets is unknown.

Diagnosis is based on clinical, histological, and immunochemical criteria. If untreated, pemphigus has a poor prognosis, and mortality is high, especially for PV. Treatment is primarily by systemic corticosteroids, and adjuvant broad-scale immunosuppression, whose side effects can be severe. Other adjuvant therapies for patients with high levels of circulating autoantibodies are high-dose intravenous immunoglobulin (IVIg) and plasmapheresis or extracorporeal immunoadsorption with protein A. A promising option is the depletion of B lymphocytes with rituximab, a monoclonal antibody targeting CD20+ B cells, particularly in the treatment of patients who develop serious side effects or do not respond to conventional therapy. Some emerging therapies that have shown positive outcomes in other autoimmune diseases are being investigated (Ruocco *et al.*, 2013; Kasperkiewicz *et al.*, 2017; Hans-Filho *et al.*, 2018; Yanovsky *et al.*, 2019).

Pemphigus frequency varies according to geographic area and ethnic groups (Alpsoy *et al.*, 2015). Both PF and PV are rare, but, in most of the world, PV is more frequent, corresponding to 65% - 85% of the pemphigus cases. The mean incidence of PV usually is higher in women, but the female:male ratio varies among populations from 0.45 to 5. The incidence reported for the different regions of Europe ranged between 0.5 and 2.4 per million and year. In Southern and Eastern Europe, the frequency of the disease is higher than in North and Central Europe; in Turkey, the yearly incidence was reported as 2.4 per million. The incidence in Asia varied between 1.6 and 16.1 per million. In North America, the incidence was reported as 32 per million in people of Jewish origin and 4.2 per million for people on non-Jewish ancestry. In Africa, the yearly incidence of pemphigus was reported as 2.9 per million in Mali and 6.7 and 8.6 per million in Tunisia. Most of these figures refer to pemphigus in general, or to only PV.

As for PF, it is generally even rarer than PV, but PF reaches high frequency in regions of endemicity in South America and Tunisia. The highest incidence of PF occurs in central-western Brazil, where the disease is known as *fogo selvagem* (FS, meaning wild fire in Portuguese). The incidence varies among regions and time from 9 to 83 cases per million inhabitants per year and the female:male ratio is approximately 1.5. Endemic foci have also been reported for Colombia, Venezuela, Peru, Bolivia, Argentina and Paraguay (Chiossi and Roselino, 2001). The highest prevalence has been observed in the Xavante and Terena Amerindians (1.4% and more than 3%, respectively; Aoki *et al.*, 2004). In central and southern Tunisia, the yearly incidence of pemphigus was estimated at 6.7 cases per million and year, of which 61% were PF, particularly women living in rural areas, with a female:male ratio of 4.1 (Bastuji-Garin *et al.*, 1995). However, in the north of the country, the incidence was of 8.6 cases per million and 61% were PV patients with a female:male ratio of 2 (Zaraa *et al.*, 2011).

**Figure 1:**
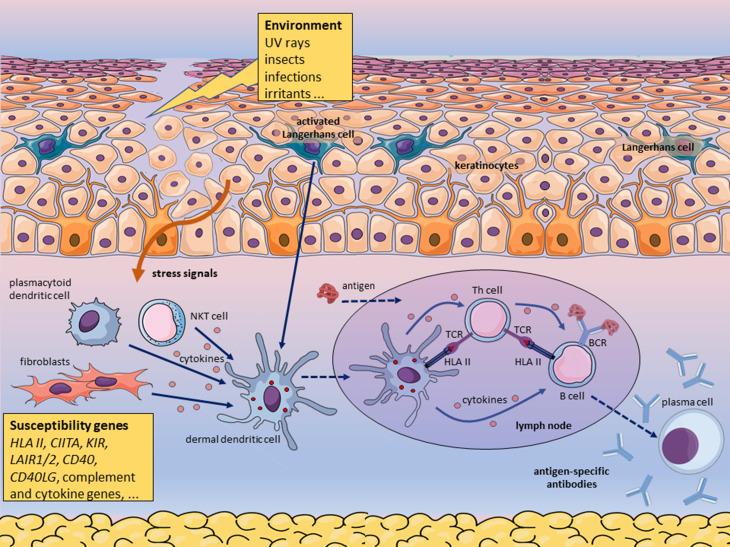
Environmental factors trigger pemphigus in genetically predisposed individuals carrying susceptibility genotypes. According to this hypothetical mechanism, insect saliva, virus, or other environmental factor triggers mast cell degranulation, which increases the permeability of blood vessels, causing edema. Langerhans cells and keratinocytes react to the noxious environmental stimulus producing pro-inflammatory cytokines and delivering other stress signals. Skin-resident innate immune cells and fibroblasts may contribute to the local inflammatory response. Inflammation leads to the recruitment of neutrophils and monocytes. The first encountered antigen derived from the environmental triggering factor is yet unknown. Activated antigen-presenting cells (APC) such as dermal dendritic cells process proteins derived from the environmental agent, migrate to skin-draining lymph nodes and present antigenic peptides bound to HLA class II molecules to T cells. In the secondary lymphoid organ, the T helper cells activate B cells primed by the same antigen. This model predicts that T cells specific for an environmental peptide bound to a susceptibility HLA class II molecule cross-reacts with a self-peptide bound to the same type of HLA molecule, such that peptides derived from a non-self protein mimic peptides of the self-protein desmoglein when bound to the relevant HLA protein. Similarly, the disease may be triggered in the intestine and perhaps other tissues by an environmental or microbiota-derived antigen.

The mechanism resulting in the breakdown of the immunological tolerance remains unknown. However, it seems settled that the onset and the course of pemphigus depend on environmental factors triggering the disease in individuals with a predisposing genetic background (Figure 1). Although essential, the complex genetic background does not suffice for disease outbreak; exposure to ill-defined precipitating environmental factors is required. These also may differ between subjects and are related to their lifestyle. Many factors have been associated with the onset or the course of pemphigus. Certain drugs may interfere with the keratinocyte membrane biochemistry and/or with the immune balance (respectively, biochemical and immunologic acantholysis). Viral infections, primarily the herpetic ones, may trigger the outbreak of pemphigus or complicate its clinical course. The precipitating effect of the viral attack may result from overactivated inflammatory and immune responses. Rare, but well-documented events that may trigger the disease in susceptible individuals are physical agents (ultraviolet or ionizing radiation, thermal or electrical burns, surgery and cosmetic procedures), contact allergens (e.g., organophosphate pesticides), dietary factors (e.g., garlic, leek, onion, black pepper, red chili pepper, red wine, tea), and emotional stress (Ruocco *et al.*, 2013). Epidemiological features of FS in Brazil indicate continued exposure to certain hematophagous insect bites as a possible precipitating factor of the disease (Lombardi *et al.*, 1992; Aoki *et al.*, 2004; Qian *et al.*, 2016).

Herein I provide a comprehensive overview of the genetic risk factors and discuss insights into pemphigus pathogenesis that this knowledge is revealing. Both pemphigus foliaceus and pemphigus vulgaris are addressed. Associations are discussed considering the function of the gene product, the allele or haplotype frequencies in populations, the strength and statistical significance of the association, sample size, and statistical power.

## Pemphigus genetics

### Evidence for a genetic basis

Although no systematic study of pemphigus recurrence in families has been published, several reports underscore the influence of a polygenic genetic background in susceptibility and pathogenesis.

Among first-degree relatives of PV patients only two cases (0.24%) of PV were seen among 830 first-degree relatives of PV patients, and none among relatives of 890 controls, while the prevalence of any autoimmune disease (AD) was 7.4% in relatives of the patients compared to 2.3% in relatives of the controls (Firooz *et al.*, 1994). In a survey of 171 PV patients, the prevalence of AD among first-, second-and third-degree relatives of patients was 50.6%, and 34.3% and 15.1%, respectively (Gupta *et al.*, 2011), in agreement with partially shared genetic and environmental factors between AD.

In one Terena Amerindian population in Brazil with a prevalence of FS close to 3%, over half of the patients (16 of 29) had at least one relative (parent, sibling, aunt/uncle, cousin) with the disease (Hans-Filho *et al.*, 1999). Unfortunately, the prevalence among matched relatives of non-diseased individuals in that population was not reported.

The wide range of incidence among populations around the globe is often interpreted as evidence for a genetic basis of multifactorial diseases such as pemphigus, however (differently from monogenic disease), variable exposure to triggering non-genetic factors is a likely cause of this heterogeneity. The alternative hypotheses should be better explored in future studies.

Nonetheless, the associations with multiple genetic variants thus far described support the hypothesis that pemphigus are complex multifactorial diseases. Susceptibility is clearly polygenic, meaning that specific genotypes at multiple loci are involved. As is the case for other complex disorders, the involved polygenes overlap only partially between patients and none of the variants conferring genetic susceptibility is essential or sufficient for disease manifestation.

Variants of numerous genes have been analyzed, especially in FS, almost always in case-control association studies. Most candidates were genes whose products are involved in immune responses, in line with the autoimmune and auto-inflammatory features of pemphigus. Data for PV and PF exist for populations of Europe, South and North America, East Asia, the Middle East, and North Africa and are described below and summarized in Table 1, and Tables S1 and S2.

**Table 1:** Non-HLA and non-classical HLA gene polymorphisms associated with differential susceptibility to pemphigus.

Disease	Gene symbol	Gene product	Location	Variant, marker, haplotype or genotype	Susceptibility or Protection?	Comments	Country or Population	References
PV	*HLA-E*	Major Histocompatibility Complex, Class I, E	6p22.1	genotype 01:03/01:03	susc	Not due to LD with HLA DRB1 or DQB1	USA	Bhanusali *et at.,* 2012
PV	*HLA-G*	Major Histocompatibility Complex, Class I, G	6p22.1	rs371194629 (14bp del)	susc		Israel Ashkenazi Jews	Gazit *et al*.,2004
EPF	*HSPA1L*	Heat Shock Protein Family A (Hsp70) Member 1 Like	6p21.33	rs2227956 C>T (Thr493Met) allele T	susc		Tunisia	Toumi *et at.*, 2015
EPF	*HSPA1A*	Heat Shock Protein Family A (Hsp70) Member 1A	6p21.33	rs1043618G > C (a 5' UTR SNP) genotype C/C	susc		Tunisia	Toumi *et at.*, 2015
EPF	*HSPA1B*	Heat Shock Protein Family A (Hsp70) Member IB	6p21.33	rs1061581 G > A (a synonymous variant) genotype G/G	susc		Tunisia	Toumi *et at*., 2015
PV	*TAP1, TAP2*	Transporters 1 and 2, ATP Binding Cassette Subfamily B Member	6p21.32	polymorphic amino acid residue frequencies	susc		Israeli Jews	Slomov *et al*., 2005
PV + PF	*TAP1, TAP2*				none		Japan	Niizeki *et al*., 2004
FS	*MHC2TA (CIITA)*	Class II Major Histocompatibility Complex Transactivator	16p13.13	rs3087456 G/G plus G/A genotypes	susc	Strong additive interaction between MHC2TA rs3087456 and HLA-DRB1 genotypes. No association with rs4774 (Gly500Ala)	Brazil general population	Piovezan and Petzl-Erler, 2013
PF, EPF. (FS)	*DSG1*	Desmoglein 1	18q12.1	allele C (especially in genotype C/C) of the synonymous SNP rs12967407 (809T > C)	susc	ns for FS (p=0.079). Interaction between DSG1 and HLA was observed by Martel *et at* (2002). Haplotypes of rs8091003, rs8091117, rs16961689, rs61730306, rs34302455 not associated	France, Tunisia, Brazil	Martel *et at.,* 2001; *Ayed et at., 2002*; Petzl-Erler and Malheiros, 2005
PV	*DSG3*	Desmoglein 3	18q12.1	Two related haplotypes were associated	susc	Association with the haplotype possibly due to an (additional) regulatory SNP (Capon *et al.*, 2009)	British and Indian populations	Capon *et al.,* 2006
FS	*KIR*	Killer Cell Immunoglobulin Like Receptor gene complex	19q13.42	More than three activating KIR and higher ratios activating/inhibitory KIR. Presence of both the activating KIR3DS1 gene and its HLA-Bw4 ligand	prot		Brazil general population	Augusto *et al.,* 2012
FS	*KIR3DL2*	Killer Cell Immunoglobulin Like Receptor KIR3DL2	19q13.42	Allele KIR3DL2*001; rs3745902 allele T (376Met)	susc; prot	The risk was higher for KIR3DL2*001/001 homozygotes than for 001/x heterozygotes; the risk was higher for presence of KIR3DL2*001 together with the ligands HLA-A3 or HLA-A11 than for presence of 001 in the absence of these KIR3DL2 ligands. SNP rs3745902 T (376Met) associated with reduced risk	Brazil general population	Augusto *et al.,* 2015
FS	*LAIR1*	Leukocyte Associated Immunoglobulin Like Receptor 1	19q13.42	rs56802430 allele G; rs11084332 allele C	susc; prot	Alleles of four SNPs mark increased mRNA expression: rs3826753 G, rs74463408 C, rs3745444 T, rs56802430 G. However, no link between LAIR1 expression and the disease was observed	Brazil general population	Camargo *et at.,* 2016
FS	*LAIR2*	Leukocyte Associated Immunoglobulin Like Receptor 2	19q13.42	rs2287828 allele T; haplotype G-T-C-A of rs2042287, rs2287828, rs2277974, rs114834145	susc	The G-T-C-A haplotype is associated with both FS and higher LAIR2 mRNA levels	Brazil general population	Camargo *et al.,* 2016
FS	*KLRG1*	Killer Cell Lectin Like Receptor G1	12p13.31	rs1805672 G allele (A/G genotype)	susc	rs1805672*G allele a miR-584-5p binding site in the 3' UTR of KLRG1	Brazil general population	Cipolla *et al*., 2016
FS	*IL6*	Interleukin 6	7p15.3	rs1800795 (-174G>C) C/C genotype	prot	rs1800795 is in the gene promoter	Brazil general population	Pereira *et al.,* 2004
FS, EPF	*IL4*	Interleukin 4	5q31.1	rs2243250 (also known as −590C > T or −589C > T) genotype T/T	susc	rs2243250 is in the gene promoter	Brazil, Tunisia	Pereira *et al.,* 2004, Toumi *et al.,* 2013
EPF	*IL4* + *IL4R*	Interleukin 4, Interleukin 4 Receptor	5q31.1, 16p12.1	T;A-C-A combination for rs2243250 (of IL4), and rs4787948-rs3024622-rs3024530 (of IL4R)	susc		Tunisia	Toumi *et at.,* 2013
EPF	*IL23R*	Interleukin 23 Receptor	1p31.3	rs11209026 G/G genotype	susc		Tunisia	Ben Jmaa *et al.*, 2018
EPF	*IL17A*	Interleukin 17A	6p12.2	rs3748067 C/C genotype	susc		Tunisia	Ben Jmaa *et al.*, 2018
EPF	*IL17F*	Interleukin 17F	6p12.2	rs763780 C allele	susc		Tunisia	Ben Jmaa *et al.*, 2018
EPF	*TNF*	Tumor Necrosis Factor	6p21.33	rs1800629 (-308G>A) A allele (in both the A/A and A/G genotypes)	susc		Tunisia	Ben Jmaa *et al.*, 2018
FS	*CD40LG* #	CD40 Ligand	Xq26.3	rs3092945 (-726T > C) allele T	susc	No association was seen for the 3'UTR(CA) STR rs56074249	Brazil general population	Malheiros and Petzl-Erler, 2009
FS	*CD40* #	CD40 Molecule	20q13.12	rs1883832 (-1C>T) allele T	prot	rs1883832 is in the Kozak sequence that includes the translation initiation codon (AUG) and is important for ribosome binding to the mRNA	Brazil general population	Malheiros and Petzl-Erler, 2009
FS	*TNFSF13B* # *(BAFF; BLYS)*	TNF Superfamily Member 13b	13q33.3	rs9514828 SNP (-871C> T) allele T	prot	rs9514828 is in the binding site for transcription factor MZF1 and may change its affinity, resulting in altered levels of BAFF	Brazil general population	Malheiros and Petzl-Erler, 2009
FS	*CTLA4*	Cytotoxic T-Lymphocyte Associated Protein 4	2q33.2	rs5742909 (-318C>T) allele T	prot	8 SNPs and 3 STR were analyzed, ranging from the promoter region of the CD28 gene to the intergenic region between CTLA4 and ICOS. rs5742909*T marks increased expression of CTLA4, which could lower the risk of Ads	Brazil general population	Dalla-Costa *et al.,* 2010
FS	*CTLA4*	Cytotoxic T-Lymphocyte Associated Protein 4		rs733618 (-1722TC) allele C	susc	rs733618*C might lead to altered alternative splicing and decreased expression and function of membrane-bound CTLA4	Brazil general population	Dalla-Costa *et at.,* 2010
FS	*CD86*	CD86 Molecule	3q13.33	rs1129055 (1057G> A, Ala304Thr) allele A	prot	rs1129055*A may alter the signal transduction pathways controlled by CD86 on antigen presenting cells. Association was significant in the sample of predominantly African ancestry, but not in Euro-Brazilians	Brazil general population	Dalla-Costa *et al.,* 2010
FS	*PDCD1 (PD-1)*	Programmed Cell Death 1	2q37.3	*rs10204525* (PD1.6) allele A	susc	rs10204525 may influence binding of microRNA and transcription factors. Association was reported for the sample of European but not of predominantly African ancestry	Brazil general population	Braun-Prado and Petzl-Erler, 2007
PV + PF	*ICOS*	Inducible T Cell Costimulator	2q33.2	rs10932029 (IVS1+173T> C) allele C	susc		Poland	Narbutt *et al*., 2010
FS	*CD59*	CD59 Molecule (CD59 Blood Group)	11p13	rs1047581 and other five SNPs. Haplotype G-G-C-C-A-A	susc	6 SNPs that might affect alternative splicing or mRNA stability were analyzed. Haplotype G-G-C-C-A-A also marks increased CD59 expression	Brazil general population	Salviano-Silva *et al.,* 2017
FS	*C3*	Complement component C3	19p13.3	rs4807895 allele T	susc		Brazil general population	Bumiller-Bini *et al.,* 2018 analyzed 992 SNPs of 44 complement system genes. Polymorphisms of 25% of them were associated with PF.
FS	*C5AR1*	Complement C5a Receptor 1	19q13.32	rs10404456 allele C	susc		Brazil general population	Bumiller-Bini *et al.,* 2018
FS	*C8A*	Complement C8 Alpha Chain	1p32.2	rs11206934 allele C	susc		Brazil general population	Bumiller-Bini *et al.,* 2018
FS	*C9*	Complement component C9	5p13.1	rs700218 allele T	prot		Brazil general population	Bumiller-Bini *et al.,* 2018
FS	*C9*			rs187875 allele T	susc		Brazil general population	Bumiller-Bini *et al.,* 2018
FS	*CFH*	Complement Factor H	1q31.3	rs34388368 genotype T/T	susc	rs34388368*T is associated with higher CFH mRNA levels in the hypodermis	Brazil general population	Bumiller-Bini *et al.,* 2018
FS	*CR1*	Complement C3b/C4b Receptor 1 (Knops Blood Group)	1q32.2	haplotype with rs6656401	susc		Brazil general population	Oliveira *et al*., 2019
FS	*CR2*	Complement C3d Receptor 2	1q32.2	rs2182911 allele C	prot		Brazil general population	Bumiller-Bini *et al.,* 2018
FS	*ITGAM (CR3)*	Integrin Subunit Alpha M / Complement Component 3 Receptor 3 Subunit	16p11.2	rs12928810 allele A	prot		Brazil general population	Bumiller-Bini *et al.,* 2018
FS	*ITGAX (CR4)*	Integrin Subunit Alpha X / Complement Component 3 Receptor 4 Subunit	16p11.2	rs11574637 allele C	prot		Brazil general population	Bumiller-Bini *et al.,* 2018
FS	*MASP1*	Mannan Binding Lectin Serine Peptidase 1	3q27.3	rs13094773 genotype G/G; rs850309G/G;rs72549154 (Arg576Met) allele T	prot	higher MASP-3 levels may lower the PF risk	Brazil general population	Bumiller-Bini *et al.,* 2018
FS	*MASP1*			rs3864098 allele C; rs698104 allele T	susc	higher MASP-1 levels may contribute to PF	Brazil general population	Bumiller-Bini *et al.,* 2018
PV + PF	*FCGR2B*	Low Affinity Receptor IIb for Fc Fragment of IgG	1q23.3	rs3219018 (-386G>C) allele C	prot	rs3219018 is shared by the promoters of FCGR2C and FCGR2B and leads to higher expression levels of FcgRIIb	Germany	Recke *et al.,* 2015
PV + PF	*FCGR2C*	Low Affinity Receptor IIc for Fc Fragment of IgG	1q23.3	rs183547105 (an ORF/Stop polymorphism) ORF allele	susc	FCGR2C expression occur only in the presence of the ORF allele. FcgRIIb expression was increased by the presence of FCGR2C ORF. The inhibitory FcgRIIb is involved in tolerance of B lymphocytes, which may be counterbalanced by FcgRIIc expression	Germany	Recke *et al.,* 2015
EPF + PF	*FOXP3*	Forkhead Box P3	Xp11.23	G-A-15-C-Chaplotype of rs3761547-rs3761548-(GT)n-rs3761549-rs2294021	susc	the four SNPs mark three different LD blocks	Tunisia	Ben Jmaa *et al.*, 2017
PV	*ST18*	ST18 C2H2C-Type Zinc Finger Transcription Factor	8q11.23	rs2304365 allele A; rs17315309 allele G	susc	both SNPs present LD in Jews. Possibly rs17315309*G, which drives ST18 upregulation is the causal polymorphism. Association was not seen in Germans (Sarig *et al.,* 2012) and in Chinese (Yue *et al.,* 2014)	Israel (Jews); Egypt; Iran	Sarig *et al.,* 2012; Vodo *et al*., 2016; Etesami *et al.,* 2018
FS	*AL110292.1*	lncRNA AL110292.1	14q12	rs7144332 allele T	susc			Lobo-Alves *et al.,* 2019b. A total of 2 080 SNPs located in long ncRNAs (lncRNAs) genes were analyzed.
FS	*LINC01176*	Long Intergenic Non-Protein Coding RNA 1176	7p14.3	rs6942557 allele C	susc			Lobo-Alves *et al.,* 2019b
FS	*LINC01119*	Long Intergenic Non-Protein Coding RNA 1119	2p21	rs17774133 allele T	susc			Lobo-Alves *et al.,* 2019b
FS	*lnc-PREX1-7:1*	lncRNA lnc-PREX1-7:1	20q13	rs6095016 allele A	prot			Lobo-Alves *et al.,* 2019b
FS	*AC009121.1*	lncRNA AC009121.1	16p13.13	rs7195536 allele G	prot			Lobo-Alves *et al.,* 2019b
FS	*AC133785.1*	lncRNA AC133785.1	2q21.1	rs1542604 allele T	prot			Lobo-Alves *et al.,* 2019b

# Gene-gene interactions were observed between *BAFF* and both *CD40* and *CD40LG:* The protective effects of *CD40LG rs3092945 C* and *CD40 rs1883832 T* alleles only manifest *in BAFF rs9514828 T*-positive individuals, and vice versa.

susc, prot: respectively, increased and decreased pemphigus risk

PV: pemphigus vulgaris, PF: sporadic pemphigus foliaceus, EPF: endemic pemphigus foliaceus in Tunisia, FS: fogo selvagem, the endemic form of pemphigus foliaceus in Brazil. LD: linkage disequilibrium, lncRNA: long non-coding RNA, ns: non-significant,

### HLA and other major histocompatibility complex (MHC) genes

#### Classical HLA genes

As for most ADs, for pemphigus associations with the classical HLA class II genes were also the first described and their variants are the strongest determinants of disease risk. Since the mid-1970s, numerous studies addressed a possible effect of HLA alleles on PV and PF pathogenesis. Pioneering studies used low-resolution serology to genotype HLA class I antigens only. Soon after the development of medium- to high-resolution typing at the DNA level in the 1980s, it was thought that the “true” associations with individual alleles would be discovered, facilitating understanding of the mechanisms governing susceptibility/resistance to pemphigus and other HLA associated diseases. However, since then, the enhanced insight into protein structure vs. function revealed additional layers of complexity.

The mechanisms by which some *HLA* alleles may impact the development of ADs are not precisely known, but it is reasonable to suppose that they are related to structural and functional aspects of peptide binding and interaction with the cognate receptors. Like most proteins, the HLA molecules also have pleiotropic effects, as illustrated by the functions of HLA class I molecules as ligands for both the T lymphocyte receptors and NK cell receptors. Very different polymorphic motifs of the HLA and their receptor molecules impact these two interactions. Also, the expression levels may differ between alleles of the same HLA locus, with functional consequences. Therefore, the genetic associations with diseases shall be further investigated, considering that functional complexity.

On the other hand, there are some characteristics of HLA that hinder a clear conclusion about the causal susceptibility and protective HLA class II alleles in pemphigus as well as other diseases: (a) linkage disequilibrium (LD) between the analyzed genes, as will be discussed below; (b) additive or epistatic functional interactions between *HLADRB1, DQA1* and *DQB1,* meaning that the haplotype rather than the individual alleles correspond to the disease-relevant genetic unit. The same rationale can be extended to the HLA genotype (union of the two HLA haplotypes of any individual) and other not investigated MHC genes; (c) the multiple HLA-DQ molecules in double heterozygous individuals: the HLA-DQ heterodimer can be formed by the alpha and beta chains encoded in *cis*, i.e., by *HLA-DQA1* and *-DQB1* genes of the same haplotype, or in *trans*, the corresponding genes of the paternal plus the maternal haplotype.

In most studies, only the *HLA-DRB1* and *HLA-DQB1* class II genes were analyzed, but several also analyzed *HLA-DQA1*; a few searched for associations with the classical class I (Ia) genes *HLA-A*, *HLA-B* or *HLA-C*. Even less looked at non-classical HLA class I genes (Ib) and non-HLA MHC genes.

Regarding the class Ia genes, associations differ greatly between populations, while this is less evident for the class II genes. Probably the majority of the class Ia associations result from LD with *HLA-DRB1* and *HLA-DQB1* alleles, but a direct effect cannot be ruled out. Indeed, associations between PF and the HLA Ia ligands of KIR have been detected (Augusto *et al.*, 2012, 2015) and will be presented in this review together with *KIR* associations.

While PF and PV share several susceptibility and protective HLA variants, significant differences point to partially dissimilar etiology and pathogenesis of these diseases. Conversely, differences among populations for the same pemphigus form are mostly due to the different frequencies of the alleles and haplotypes, but some may have a biological basis, possibly related to environmental triggering factors and the immune response to specific peptides. The observed associations are described below. A summary of the associations between pemphigus and HLA class II alleles and haplotypes is presented in Tables S1 and S2.

Much work has been dedicated to the search of associations between *HLA* alleles of the classical class II genes and haplotypes in pemphigus vulgaris. Ashkenazy Jewish patients with PV presented significantly higher frequencies of *HLA-DR4-HLA-DQw8(3)* haplotypes than the matched control group (Ahmed *et al.*, 1990). The results of case-control association studies of European, Western and Southern Asian, and North and South American populations of European and Western Asian ancestry showed that the risk haplotype is DRB1*04:02-DQA1*03:01-DQB1*03:02 (Table S1). Apart from the data presented in the PV studies, this conclusion is based on the frequency of the individual alleles and this haplotype in the populations studied, available at Allele Frequencies Net Database (AFND; Gonzalez-Galarza *et al.*, 2018). Allele DRB1*04:06 in the Japanese and Chinese is a PV susceptibility allele as well (Yamashina *et al.*, 1998; Zhang *et al.*, 2019). In the DRB1*04 haplotypes, the association with DQA1*03:01-DQB1*03:02 seems secondary to the association with DRB1*04, as a result of high linkage disequilibrium (LD): The alleles DRB1*04:01, 04:03 and *04:05* (and other) also are inserted in DQA1*03:01-DQB1*03:02 haplotypes, but were not associated with increased susceptibility to PV (e.g., Carcassi *et al.*, 1999; Haase *et al.*, 2015). In fact, DRB1*04:01 may be a protective allele in the German population (Haase *et al.*, 2015).

Another haplotype commonly associated with increased PV risk is DRB1*14:54(14:01)-DQA1*01:04-DQB1*05:03 (or DQB1*05:02 in Iran and Japan) (Table S1). Before its description, allele DRB1*14:54 was routinely typed as DRB1*14:01. Resolving the DRB1*14:54/14:01 ambiguity showed high relative ratios of DRB1*14:54 (87-94%) in north to central Europe, contrasting with lower ratios (47-76%) in south and east Europe (Vidan-Jeras *et al.*, 2014). In East Asia, all individuals previously typed as DRB1*14:01 were DRB1*14:54 and DQB1*05:02 or *05:03* (Lee and Jung, 2009; He *et al.*, 2012). The functional impact of this difference – that is limited to one amino acid replacement at position 112 – may not be relevant for *HLA-DRB1* biological function. Other DRB1*14 alleles also are associated with PV risk: In European and Euro-descendant populations, DRB1*14:04 and *14:05, which occur in DQB1*05:03 haplotypes (Table S1). In Indo-Asians DRB1*14:04 also is associated with PV (Saha *et al.*, 2010). In Japanese, the haplotypes DRB1*14:54(14:01) or 14:05-DQA1*01:04-DQB1*05:03 were associated with PV (Yamashina *et al.*, 1998). In Chinese, DRB1*14:04, 14:05, and *14:54* were common among PV patients, as well as DQA1*01:01 and DQB1*05:03 (Sun *et al.*, 2019; Zhang *et al.*, 2019); however, haplotype analysis was not performed for that population. In fact, all the DRB1*14 alleles that are common in one or the other of the populations studied (references in Table S1 and Gonzalez-Galarza *et al.*, 2018) were associated with increased PV risk. Differences between populations are mostly explained by the allele frequencies and/or low statistical power. It has been suggested that the disease-relevant allele of the DRB1*14:54(14:01) haplotype is DQB1*05:03 alone (Lee *et al.*, 2009), but the PV risk haplotype DRB1*14:54/14:01-DQB1*05:02 haplotype (Yamashina *et al.*, 1998; Shams *et al.*, 2009) challenges this interpretation. Conversely, allele DQB1*05:02 also occurs in the non-susceptibility DRB1*15 and DRB1*16-bearing haplotypes (Párnická *et al.*, 2013; Zivanovic *et al.*, 2016) and cannot be a causal susceptible or protective allele.

Moreover, the allele DRB1*08:04 is associated with PV in Brazilian and Egyptian (DRB1*08:04-DQA1*05:01-DQB1*03:01), and Italian (possibly DRB1*08:04-DQA1*04:01-DQB1*04:02) populations. The 3-loci haplotypes were inferred here based on the PV-associations (Table S1) and worldwide frequencies available at AFND (Gonzalez-Galarza *et al.*, 2018).

Several of the studies do not report protective alleles and haplotypes or mention only the allele groups. The available data indicate that all major *HLA-DRB1* allele groups apart from DRB1*04, 08 and *14* present lower frequency among PV patients compared to controls: DRB1*03, 07, 11, 13 and *15* in six to eleven of the populations DRB1*01 in three, DRB1*16 in two, and DRB1*09 in one (Table S1). This difference between associations of PV with *DRB1* alleles is mostly due to low allele frequency in the populations studied. For example, the frequency of DRB1*09 is of 0 to 2% in the populations with exception of Chinese and Japanese (12% - 16%). For HLA-DQB1, alleles DQB1*02, *06 and *03:01 are markers of decreased risk (Table S1). The LD pattern indicates that HLA-DQB1 rather than HLA-DRB1 alleles may be the relevant protective factors: Worldwide, DQB1*02 occurs almost exclusively together with DRB1*03:01 or *07:01* whose frequency is decreased among patients*.* However, DRB1*07:01 may also occur with DQB1*03:03 or *05:01* (Gonzalez-Galarza *et al.*, 2018) which are not associated with PV. Similarly, alleles of group DQB1*06 occur in haplotypes bearing *HLA-DRB1* alleles that belong to the DRB1*13 and *15* groups. Moreover, structural differences between DRB1*03:01 and *07:01*, and between the DRB1*13 and 15 are great, resulting in distinct peptide-binding properties. For these reasons, DQB1*02 and DQB1*06 most likely are the protective alleles in the corresponding haplotypes. By contrast, allele DQB1*03:01, also associated with low PV risk occurs in both protective (DRB1*11), and susceptibility haplotypes (DRB1*08:04) and cannot have a direct effect on PV.

As to pemphigus foliaceus, in the Brazilian population, increased susceptibility to FS is associated especially with alleles of the HLA-DRB1*01 and DRB1*04 groups (Petzl-Erler and Santamaria, 1989). The *HLA-DRB1* alleles associated with highest risk are DRB1*01:02 and *04:04*;additional significant associations occur with DRB1*01:01, 01:03, 04:06, *14:06, 16:01* (Moraes *et al.*, 1997; Pavoni *et al.*, 2003). Low risk alleles are DRB1*07:01, 03:01, 14:02, the allele group *08* (encompassing alleles *08:01, 08:02, 08:03, 08:04* and *08:07* in that population), group *11 (11:01, 11:02, 11:03, 11:04)*, and group *15* (alleles *15:01, 15:02, 15:03*) (Table S2). It has been suggested that increased susceptibility could be associated with the motif LLEQRRAA of the amino acid residues 67-74 (Cerna *et al.*, 1993; Moraes *et al.*, 1997). However, a subsequent study concluded that HLA-DRB1 protein motifs do not add much to comprehension of the molecular basis of the *HLA-DRB1* - FS associations (Pavoni *et al.*, 2003).

**Figure 2:**
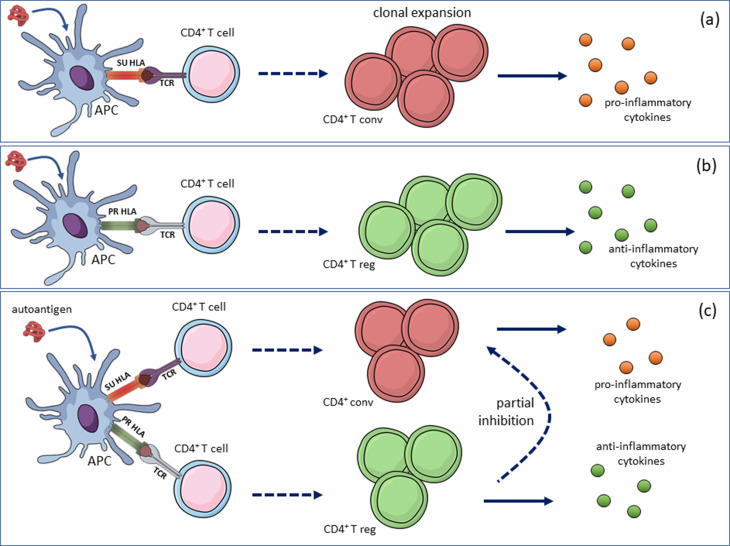
Incompletely dominant effect of protective HLA alleles by antigen-specific regulatory T cells. When HLA class II alleles were grouped in three categories, susceptibility (SU), protective (PR), and neutral (NE), Pavoni *et al.* (2003) observed that SU/SU and SU/NE were susceptibility genotypes (a), while the PR/PR and PR/NE genotypes were highly protective (b); conversely, the PR/SU genotype resulted in a neutral phenotype, similar to that of NE/NE. According to the model shown, the partial inhibition of the autoagressive response by conventional effector T cell in individuals with a SU/PR genotype is provided by the anti-inflammatory response of regulatory T cells (model based on Ooi *et al.*, 2017). APC: antigen-presenting cell, TCR: T cell receptor, T conv: conventional effector T cell, T reg: regulatory T cell.

When *HLA-DRB1* alleles were grouped in three categories, susceptibility (SU), protective (PR), and neutral (NE), an additive effect of SU was observed: the risk for SU/SU genotypes was about twice the risk for SU/NE genotypes; the PR/PR and PR/NE genotypes were equally highly protective; conversely, the PR/SU and NE/NE genotypes exhibited a neutral phenotype (Pavoni *et al.*, 2003). A dominant effect of protective HLA alleles has been reported for other autoimmune diseases and may result from the action of autoantigen-specific regulatory T lymphocytes (Treg cells) (Ooi *et al.*, 2017) (Figure 2)

As to *HLA DRB1-DQA1*-*DQB1* haplotypes, increased risk was observed for DRB1*01-DQA1*01:01-DQB1*05:01. Decreased risk haplotypes were DRB1*07:01-DQA1*02:01-DQB1*02:01, DRB1*03:01-DQA1*05:01-DQB1*02:01, and DRB1*11-DQA1*05:01-DQB1*03:01 and DRB1*14:02-DQA1*05:01-DQB1*03:01 (Pavoni *et al.*, 2003; Brochado *et al.*, 2016) and DRB1*15-DQB1*06:02 (Moraes *et al.*, 1991; Pavoni *et al.*, 2003). It is difficult to identify the primary association or detect additive or epistatic gene-gene interactions because *HLA-DQ* and *HLA-DRB1* alleles present high LD. However, the most constant markers of high risk are DQB1*05 and *03:02*, while DQB1*02, 03:01 and *06* mark most low risk haplotypes (Table S2).

In Amerindian populations, a different picture emerges. In both the Xavante and the Terena populations, the FS susceptibility *HLA-DRB1* allele is *04:04*, while DRB1*08:02 is associated with relative protection from the disease. An additional association with DQB1*03:02 was seen in the Terena, which agrees with its high LD with DRB1*04:04 (Cerna *et al.*, 1993; Moraes *et al.*, 1997).

These results show that the most FS-HLA associations differ between Amerindians and the analyzed general Brazilian population (which is admixed but of predominantly European ancestry). The differences arise from the contrasting *HLA* allele frequencies between these populations. South American Amerindians do not have alleles of groups DRB1*01, DRB1*03, DRB1*04:06, DRB1*07, DRB1*11, DRB1*15, DRB1*16:01, DQA1*01, DQA1*02, DQB1*02, DQB1*05, DQB1*06, or present one or the other of these alleles at low frequency due to gene flow from non-Amerindians (Tsuneto *et al.*, 2003, Gonzalez-Galarza *et al.*, 2018).

In Tunisia, PF is endemic in the south and sporadic in the north. The analysis of *HLA-DRB1* and *DQB1* revealed some differences between south and north Tunisia, and between PF in Brazil and in Tunisia. Increased susceptibility was associated with allele groups DRB1*03 (south Tunisia) and *04* (in the whole sample), and haplotypes DRB1*04:04-DQB1*03:02 (whole sample) and DRB1*04:02-DQB1*03:02 (north). Conversely, relative protection from PF was associated with the allele groups and haplotypes DRB1*11-DQB1*03:01 (south), DRB1*13-DQB1*06 or 03 (whole sample), and DRB1*15-DQB1*06 (south) and DRB1*04:05-DQB1*03:02 (in north Tunisia) (Abida *et al.*, 2009) (Table S2).

Most differences between Brazilians and Tunisians for PF-associated HLA class II alleles and haplotypes may be due to differing allele/haplotype frequencies, sample sizes, and statistical power. However, some may have a biological cause. The allele group HLA-DRB1*03 is not associated with FS, for which the two DRB1*03-bearing haplotypes have opposing effects on susceptibility (Table S2). So, allele DRB1*03:01, associated with lower FS risk in Brazil has been suggested to increase susceptibility to PF in Tunisia. The *DQA1-DQB1* haplotypes associated with DRB1*03 alleles are the same in Brazil and Tunisia (Gonzalez-Galarza *et al.*, 2018) and therefore cannot be the cause of the observed difference. Haplotypes that are common also in Tunisia but seemingly have no effect on PF susceptibility in that population are DRB1*07:01-DQA1*02:01-DQB1*02:01 or 03:01 and DRB1*01-DQA1*01:01-DQB1*05:01, respectively associated with low and high risk of FS in Brazil. The discrepancy between the Tunisian and Brazilian populations may at least partially stem from different environmental triggering factors, or from differential LD with other not analyzed relevant MHC genes.

For sporadic PF in China (Sun *et al.*, 2019; Zhang *et al.*, 2019) the high risk markers were DRB1*04:06, 14:05 and DQB1*03:02, 05:03. In a small sample of patients in France, DRB1*01:02, 04:02, 04:06, 14 and DQB1*03:02 are high risk markers; DQB1*02 is associated with decreased risk (Martel *et al.*, 2002) (Table S2). Most differences between sporadic PF on these populations and endemic PF in Brazil and Tunisia probably result from the distinct allele frequencies and statistical power.

#### Other MHC genes

The MHC is a region of about 4 Mb at the cytogenetic location 6p21.33 that contains numerous genes distributed over three regions: class I, class III and class II. The MHC gene set includes classical and non-classical *HLA* class I and II genes and many additional genes whose products perform immune-related and unrelated functions.

##### • Non-classical HLA genes

Apart from HLA-A, B, C, DR, DQ, DP, all other HLA molecules are grouped as “non-classical”. There are some noteworthy differences between the classical and non-classical HLA proteins and their genes, regarding functions, expression, and polymorphism (refer to O'Callaghan and Bell, 1998; Mellins and Stern, 2013).

HLA-E is expressed on the surface of virtually every normal cell and plays a dual immunoregulatory role in innate and adaptive immune responses. It may present pathogen-derived sequences, which elicit specific T lymphocyte responses, but the best-known function of HLA-E is the modulation of NK cell responses. HLA-E binds peptides derived from the signal sequence of HLA Ia molecules, mediating inhibitory signals via the CD94-NKG2A receptor, or activating signals via the NKG2C receptor when the HLA-G leader peptide is bound to HLA-E. So, it indirectly signals HLA class I expression, protecting healthy cells against lysis by NK cells, or allowing lysis of infected cells by NK cells when HLA Ia expression is abnormally low or absent, and HLA-G is upregulated (Lauterbach *et al.*, 2015). Thus, HLA-E mediates self/non-self discrimination by NK cells, and this balance may be disturbed in pemphigus and other ADs.

In a case-control study of North American subjects, the frequency of homozygous *HLA-E 01:03/01:03* individuals was significantly increased among PV patients. The data indicate that this association did not result from LD with PV-associated *HLA-DRB1* and -*DQB1* alleles (Bhanusali *et al.*, 2013). The E*01:03 allele may increase susceptibility to other ADs as well. In a case-control study of rheumatoid arthritis (RA) in Poland, females (but not males) with the E*01:03 allele were at higher risk, and *01:01/01:01* homo-zygotes were at lower disease risk. Also, patients bearing the *01:01/01:01* genotype achieved a significantly better outcome of anti-TNF treatment than patients with the E*01:03 allele (Iwaszko *et al.*, 2015).

The HLA-G molecule plays an immunoregulatory, tolerogenic role and interacts with several immune cells, through the CD8, LILRB1 and LILRB2, and KIR2DL4 receptors. HLA-G presents four membrane-bound and three soluble isoforms, restricted tissue expression, and limited nucleotide variability in the coding region, but high variability in the promoter and 3' UTR, which may influence HLAG levels (Donadi *et al.*, 2011).

A significant increase of the *HLA-G* 14-bp deletion allele was observed in Jewish PV patients (Gazit *et al.*, 2004). This indel polymorphism *rs371194629* is in exon eight that specifies the 3' UTR region of the mRNA and has been implicated in posttranscriptional gene regulation and alternative splicing. In general, the 14-bp deletion allele has been associated with higher production of HLA-G, an effect that might be due to other polymorphisms in LD with *rs371194629* (Donadi *et al.*, 2011). The same and other HLA-G polymorphisms were associated with various diseases, including autoimmune disorders (Donadi *et al.*, 2011).

Altered expression of HLA-G and imbalance of its isoforms was observed in epidermal cells of PV patients, suggestiong that HLA-G may act to diminish the deleterious effects of disease-promoting T lymphocytes or contribute to the homeostatic balance of the skin at the end of inflammation (Yari *et al.*, 2008).

##### • Heat shock proteins of the HSP70 family

One of the first recognized functions of heat shock proteins (HSPs) is to chaperone other proteins, and most of them are upregulated during stressful conditions. Moreover, extracellular HSPs participate in the induction of cellular immune responses since they are involved in the antigen processing and presentation (de Jong *et al.*, 2014). HSP70s are one of the most abundant sources of HLA class II ligands. Natural autoantibodies to HSP70s are common, and epitopes of HSP70s are recognized by Treg cells. However, exacerbated effector responses to HSP70s are associated with ADs. These findings demonstrate a complex relationship between autoimmunity and AD: natural autoimmunity to HSP70 is associated with health, whereas altered autoimmunity to HSP70 is related to disease. In this way, HSP70 could be essential autoantigens in balancing the healthy immune system (de Jong *et al.*, 2014).

Three HSP70 family genes - *HSPA1L*, *HSPA1A*, and *HSPA1B* (often called *HSP70-1, HSP70-2* and *HSP70-HOM*, respectively) - are located in the MHC class III region. In a case-control and family study of PF in Tunisia with three tagging SNPs, increased frequencies of *HSPA1L rs2227956 C>T* (Thr493Met) allele *T*, *HSPA1A rs1043618 G>C* (a 5' UTR SNP) genotype *C/C*, and *HSPA1B rs1061581 G>A* (a synonymous variant) genotype *G/G* were observed among the patients in comparison to the control group. However, the significant LD between the *HSP70* SNPs and the *HLA* class II alleles, together with the results of the multivariate regression analysis could argue against a direct role of the *HSP70* polymorphism in susceptibility to PF (Toumi *et al.*, 2015).

##### • Conflicting results for association between pemphigus and the transporter associated with antigen processing (TAP) genes.

TAP is a heterodimeric membrane molecule of the endoplasmic reticulum (ER) required for the transportation of peptides generated by the proteasome from the cytosol to the ER lumen, where they are loaded onto the HLA class I molecules. The *TAP1* and *TAP2* genes are located between the *HLA-DQ* and *HLA-DP* genes in the MHC. In a Japanese sample, the allele, haplotype and amino acid residue frequencies at each dimorphic site did not differ between PV and PF patients and controls, nor between patients grouped according to anti-DSG autoantibody profiles (Niizeki *et al.*, 2004). However, in Israeli Jews, significant differences between PV patients and controls were detected in TAP2 polymorphic amino acid residue frequencies (Slomov *et al.*, 2005).

### Variants of the *MHC2TA (CIITA)* gene indicate that the quantitative variation of MHC class II molecules also influences susceptibility

The MHC2TA (also known as CIITA or C2TA) molecule is the master regulator of constitutive and IFNγ-induced expression of HLA class II genes in antigen-presenting cells. Mutations in the *MHC2TA* gene (cytogenetic location 16p13.13) are responsible for the bare lymphocyte syndrome (BLS), type II, complementation group A (OMIM #209920), a severe immunodeficiency in which patients fail to produce HLA class II molecules. Like several other immunodeficiencies, BLS also is often associated with autoimmune disorders. Patients have decreased numbers of Treg cells and fail to counterselect autoreactive mature naive B lymphocytes, suggesting that peripheral B cell tolerance also depends on HLA class II – T cell receptor (TCR) interactions (Hervé *et al.*, 2007). Less detrimental variants of MHC2TA may have an impact in susceptibility to multifactorial diseases, notably HLA-associated diseases.

In a case-control study of FS, two SNPs were selected for association analysis. While the missense *rs4774* (Gly500Ala) SNP in the NACHT domain was not associated with FS, the *G* allele of *rs3087456* in the promoter region was significantly associated with increased susceptibility in both the homozygous *G/G* and the heterozygous *G/A* states (Piovezan and Petzl-Erler, 2013). Additionally, a strong additive interaction between *MHC2TA* and *HLA-DRB1* genotypes in FS disease susceptibility was observed: The odds ratio for individuals having two susceptibility *HLA-DRB1* alleles was 14.1 in the presence of the susceptibility *MHC2TA rs3087456 G* allele, but much lower (2.2) in the presence of the protective *MHC2TA A/A* genotype. Based on these results, the hypothesis that genetically controlled levels of MHC2TA result in differential expression of the susceptibility and protective HLA class II molecules was raised. Thus, the quantitative variation of HLA molecules, in addition to their structural variation resulting from polymorphism of the coding regions, influences the risk of an individual developing pemphigus (Piovezan and Petzl-Erler, 2013). The same polymorphism also was associated with increased susceptibility to multiple sclerosis (MS), RA, and myocardial infarction (Swanberg *et al.*, 2005).

*MHC2TA* has four promoters that control its expression in different cell types. The *rs3087456* polymorphism can affect promoter III functionality that is responsible for the constitutive expression of *MHC2TA* in B lymphocytes, which are crucial for pemphigus autoimmunity. When leukocytes were stimulated *ex vivo* with IFNγ, lower expression of both the mRNA and the protein was seen for genotype *G/G* in comparison to genotypes *A/A* and *A/G* (Swanberg *et al.*, 2005).

### Do polymorphisms of the desmoglein 1 and desmoglein 3 genes have an impact on pemphigus pathogenesis?

Four DSG genes are closely linked in chromosome 18, at the cytogenetic position 18q12.1. *DSG1* and *DSG3* encode the major autoantigens in PF and PV, respectively. Both genes are polymorphic. Several rare pathogenic variants of *DSG1* result in autosomal dominant monogenic diseases palmoplantar keratoderma I (OMIM #148700), and congenital erythroderma with palmoplantar keratoderma, hypotrichosis, and hyper-IgE (OMIM #615508). Conversely, numerous benign SNPs that could play a role in susceptibility to polygenic disease occur in *DSG1* and *DSG3*.

The question of whether genetic variants of *DSG1* could play a role in PF was addressed in studies of French, Tunisian and Brazilian populations. Two polymorphic markers were analyzed. A haplotype comprising five missense variants in LD resulting from SNPs *rs8091003*, *rs8091117*, *rs16961689*, *rs61730306*, *rs34302455* and corresponding to the extracellular domains EC4 and EC5 was not associated with PF, indicating that the structure of this portion of the molecule does not impact PF susceptibility (Martel *et al.*, 2001). Though, allele *C* of the synonymous *rs12967407* SNP at exon 7 (809T>C) was significantly more frequent in French and Tunisian PF patients than in the respective controls, especially in the homozygous *C/C* state (Martel *et al.*, 2001; Ayed *et al.*, 2002). Additionally, interaction between *DSG1* and *HLA* variants in PF susceptibility was observed by Martel *et al.* (2002). In FS the frequency of genotype *C/C* also was increased in the patient sample, but the association was not significant (p = 0.079; Petzl-Erler and Malheiros, 2005). In that context, the unusually extended and strong LD between *rs12967407* and more than 100 polymorphisms, including SNPs in regulatory regions (Ensembl, Cunningham *et al.*, 2019), is relevant and should be explored in future studies.

Significant associations between *DSG3* variants and PV have been reported. Two related haplotypes were associated with PV in the British and Indian populations (Capon *et al.*, 2006). In a follow-up study of the British sample, additional variants were examined, and the authors concluded that the association signal detected was due to other, regulatory rather than the previously examined coding SNPs (Capon *et al.*, 2009). So, as for *DSG1* in PF, the question about the role of *DSG3* polymorphisms in PV pathogenesis is still open. Characterization of desmoglein regulatory elements and functional analysis are expected to identify sequence variants affecting gene expression and disease susceptibility.

### Genes in the leukocyte receptor complex (LRC): *KIR, LAIR1, LAIR2*

The LRC on chromosome 19q13.42 comprises many genes for immunoglobulin-like cell surface receptors (Barrow and Trowsdale, 2008). It includes genes for the killer immunoglobulin-like (*KIRs*) receptors and the leukocyte Ig-like receptors (*LILRs*). The principal known ligands for both KIRs and LILRs are HLA class I molecules. Other Ig-family genes in the LRC are *LAIR1* and *LAIR2* (leukocyte-associated Ig-like receptors-1 and −2), natural cytotoxicity triggering receptor 1 (*NCR1* also named *NKp46* or *LY94*), receptor for Fc fragment of IgA (*FCAR* or *CD89*), and platelet glycoprotein VI (*GP6*), whose ligands are as diverse as immunoglobulins, viral hemagglutinins, and collagens. The LRC also contains NLR family members (*NLRP* or *NALP*, NLR family, pyrin domain-containing) that localize inside the cell and contribute for the activation of proinflammatory caspases via their participation in multiprotein complexes called inflammasomes. The impact of the LRC on complex disease susceptibility has been poorly explored, despite its evident importance in inflammation and immunity.

A genome-wide expression profiling with approximately 55,000 probes revealed that several genes in 19q13 were differentially expressed in CD4+ lymphocytes when comparing FS patients and controls, as well as between different FS clinical forms (Malheiros *et al.*, 2014). Motivated by this result, recently the whole 1.5 Mb LRC was screened in a case-control study using genotype data of 527 tag SNPs of which three were associated with differential susceptibility to FS (Farias *et al.*, 2019). The intergenic SNP *rs465169* is in a region that regulates several immune-related genes, including *VSTM1, LAIR1, LILRA3-6, LILRB2, NLRP12,* and *LENG8.* Increased risk was associated with its minor *A* allele. The *LENG8* rs35336528 and the *FCAR* rs1865097 SNPs and four haplotypes with SNPs within the *KIR3DL2/3*, *LAIR2,* and *LILRB1* were also associated with FS.

#### The killer cell immunoglobulin-like receptor (KIR) and their HLA ligands modulate susceptibility to FS

Natural killer (NK) cells belong to the family of innate lymphoid cells and are major players of innate immune responses, and also modulate adaptive immune responses. Various reports suggested a correlation of NK cell number and functional alterations with PV and other autoimmune conditions (Takahashi *et al.*, 2007; Gianchecchi *et al.*, 2018). NK cells express numerous receptors, including KIR that are also expressed in a subpopulation of cytolytic T lymphocytes.

There are inhibitory and activating KIR. The ligands for most activating KIR are unknown, but most inhibitory KIR bind HLA class I molecules. Cells with abnormally low classical HLA class I expression may escape recognition by cytotoxic CD8 T lymphocytes, but this renders these cells sensitive for NK-mediated killing. Hence, the cytotoxic response of NK cells occurs when activating signals predominate over inhibitory signals delivered by KIR-HLA (Kulkarni *et al.*, 2008).

The genomic *KIR* region in the LCR is multigenic, but the number of *KIR* genes (gene content) varies widely, from 4 to 20 among *KIR* haplotypes. Each of these *KIR* genes presents multiple alleles. That normal variation does influence complex diseases and reproduction (Augusto and Petzl-Erler, 2015).

For FS, a protective association with activating KIR genes was observed in a study of *KIR* gene content polymorphism (Augusto *et al.*, 2012). The presence of more than three activating genes apparently lowers the risk of FS significantly, and the strongest protective effect was found for higher activating/inhibitory *KIR* ratios. Furthermore, the presence of both the activating *KIR3DS1* gene and its HLA-Bw4 ligand was protective. This contrasts with other ADs, where activating *KIR* genes have been commonly reported to increase the risk. On the contrary, for infectious diseases reduced susceptibility is associated with activating *KIR* (Kulkarni *et al.*, 2008). The authors hypothesized that this unusual association for a disease with autoimmune features might be related to the environmental trigger of FS. Possibly a viral or a salivary protein inoculated by a hematophagous insect initiates the pathogenic process. Thus, a more effective immune response against the initial triggering factor, with the participation of activating KIR, may prevent the early events that initiate the pathogenic process (Augusto *et al.*, 2012).

In a subsequent investigation of *KIR3DL2* alleles in FS, increased susceptibility was associated with allele KIR3DL2*001 in an allele-dose and ligand-dependent manner: The risk was almost fourfold increased for KIR3DL2*001/001 homozygotes, and for the presence of KIR3DL2*001 together with at least one copy of the KIR3DL2 ligands HLA-A3 or HLA-A11. Moreover, a lower percentage of KIR3DL2-positive NK cells and lower expression of *KIR3DL2* at the cell surface was seen for variant T (376Met) of SNP *rs3745902* (1190C>T, Thr376Met). Amino acid 376 is in the cytoplasmic tail of the receptor and 376Met lowers the risk of FS. Because KIR3DL2 is an inhibitory receptor, lower susceptibility to FS may be due to decreased inhibitory signals within NK cells (Augusto *et al.*, 2015). These results are in line with the gene content analysis commented above (Augusto *et al.*, 2012).

#### LAIR1 and LAIR2 gene variants are involved in gene expression and susceptibility to pemphigus foliaceus

The leukocyte-associated immunoglobulin-like receptor 1 (LAIR-1, or CD305) is a collagen-binding inhibitory receptor necessary for the regulation of immune responses, expressed on most peripheral blood mononuclear cells (PBMC). The complement component C1q and collagen XVII are among the ligands of LAIR-1. LAIR-1 ligand engagement and crosslinking suppresses the function and/or differentiation of NK cells, T and B lymphocytes, dendritic cells and its precursors, and monocytes. The principal source of its secreted homolog LAIR-2 (or CD306) are T CD4+ lymphocytes. LAIR-2 functions as a natural competitor of LAIR-1 by binding the same ligands, thus restraining the inhibitory potential of LAIR-1 (Meyaard, 2008). Altered protein levels of LAIR-1 and LAIR-2 have been associated with autoimmune and inflammatory disorders, such as systemic lupus erythematosus (SLE), RA and autoimmune thyroid diseases (ATD) (see Camargo *et al.*, 2016).

In a study of genome-wide mRNA levels in FS, both the *LAIR1* and the *C1QA* (that codes for the C1q ligand) mRNA levels were increased in CD4+ T lymphocytes of patients with disseminated (generalized) FS in comparison to unaffected controls (Malheiros *et al.*, 2014).

Two of six analyzed *LAIR1* tag SNPs (*rs56802430* allele *G* and *rs11084332* allele *C*) were respectively associated with increased and decreased susceptibility to FS, and one of eight *LAIR2* tag SNPs (*rs2287828* allele *T*) was associated with increased susceptibility in a case-control analysis for FS (Camargo *et al.*, 2016). Furthermore, 4 to 5-fold increased susceptibility was seen for a haplotype of four *LAIR2* SNPs that are not in LD with each other (r^2^ ≤ 0.08; *rs2042287*, *rs2287828*, *rs2277974*, and *rs114834145*; haplotype *G-T-C-A*). Alleles of four of the *LAIR1* SNPs mark increased mRNA expression: *rs3826753 G, rs74463408 C, rs3745444 T, rs56802430 G*; however, no link between *LAIR1* expression and the disease was observed, leading to the conclusion that FS susceptibility by *LAIR1* polymorphisms is not a consequence of variable gene expression. Conversely, the same *LAIR2 G-T-C-A* haplotype is associated with both FS and 4.5-fold higher *LAIR2* mRNA levels. The authors suggested that higher levels of the LAIR-2 protein are detrimental in FS by antagonizing LAIR-1 function and exacerbating immune responses (Camargo *et al.*, 2016). Noteworthy, most *LAIR1* and *LAIR2* SNPs associated with FS or in high LD with them are in regions that present pre- or post-transcriptional regulatory features, such as chromatin modifications, regulatory RNA binding, or RNA splicing (Camargo *et al.*, 2016).

### A regulatory 3' UTR polymorphism of *KLRG1* influences susceptibility to pemphigus foliaceus

The killer cell lectin-like receptor subfamily G member 1 protein (KLRG1, alternatively MAFA, MAFAL or CLEC15A) is an inhibitory receptor expressed on the surface of mainly NK cells, and of CD4+ and CD8+ αβ T lymphocytes with an effector or effector-memory phenotype. In addition to the inhibitory KIRs that regulate NK cell function via binding of HLA class I molecules on target cells (see above), NK cells also have inhibitory receptors specific for non-HLA ligands. KLRG1 monitors the expression of E-, Nand R-cadherins on target cells, mediating missing-self recognition by binding to a highly conserved site on these classical cadherins.

The *KLRG1* gene is in the NK cell complex (NKC) in the chromosomal region 12p13. In an analysis of candidate SNPs chosen because of their putative ability to disrupt or create microRNA binding sites, increased FS susceptibility associated with the *A/G* genotype of *KLRG1 rs1805672* compared with the *A/A* genotype was seen. The *KLRG1 rs1805672 G* allele disrupts a miR-584-5p binding site in the 3' UTR of *KLRG1*; accordingly, *KLRG1* mRNA levels were significantly higher in PBMC of *G*-positive individuals in comparison to individuals with genotype *A/A*. Functional analyses indicated that allele *G* directly interferes with miR-584-5p binding, allowing for *KLRG1* mRNA (and possibly protein) accumulation, which in turn may contribute to the pathogenesis of FS (Cipolla *et al.*, 2016). Interestingly, auto-antibodies against the KLRG1 ligand E-cadherin (CDH1) were detected in sera of about half of PF patients and healthy subjects of an endemic area in Brazil, but not in healthy individuals from USA (Flores *et al.*, 2012). It remains to be tested whether a relationship between increased KLRG1 levels, KLRG1-CDH1 binding, and anti-CDH1 autoantibodies exists in pemphigus.

### Genetic variants of some cytokines and cytokine receptors have an impact on pemphigus susceptibility

Cytokines are involved in immune responses and the regulation of numerous other biological processes. Many cytokine genes are polymorphic, and that diversity may have a functional impact, reflecting on susceptibility to complex diseases.

Interleukin 6 (IL-6) is an inducer of the acute phase response and acts on immune and non-immune cells. It is involved in monocyte and lymphocyte differentiation and is required for the generation of Th17 lymphocytes. Also, IL-6 plays an essential role in the terminal differentiation of B lymphocytes into immunoglobulin-secreting cells.

A significant association with the *IL6 rs1800795* −174G>C polymorphism was found for FS, indicating that the *C/C* genotype has a protective effect, while *G* in the homozygous or heterozygous state is associated with increased susceptibility (Pereira *et al.*, 2004). The *rs1800795* SNP is within the gene promoter. The *C* allele is associated with lower plasma levels and lower *in vitro* expression in comparison to the *G* allele (Fishman *et al.*, 1998; Rivera-Chavez *et al.*, 2003). Increased levels of IL-6 have been correlated with inflammatory and AD susceptibility, activity or more severe clinical symptoms (Mihara *et al.*, 2012).

The *rs2243250* SNP (also known as −590C>T or −589C>T) in the promoter of the *IL4* gene was investigated for PF susceptibility in Brazil and Tunisia (Pereira *et al.*, 2004; Toumi *et al.*, 2013). In both studies, the *T/T* genotype was increased in the patient samples in comparison to the control samples. Interestingly, the *T/T* genotype expresses higher mean serum levels of IL-4 compared to the *C/T* and *C/C* genotypes (Toumi *et al.*, 2013). Higher IL-4 levels might be contributing to the polarization of autoreactive Th lymphocytes towards the Th2 pathway, inducing proliferation of autoreactive B lymphocytes and facilitating immunoglobulin class switching to IgG4 that is pathogenic in pemphigus. The *IL4R* gene (also known as *IL4RA*, in 16p12.1) encodes the IL-4Rα chain of the heterodimeric receptors for IL-4 and IL-13. Toumi *et al.* (2013) observed a positive association of PF in Tunisia with the *T;A-C-A* combination for *rs2243250* (of *IL4*), and *rs4787948–rs3024622–rs3024530* (of *IL4R*), raising the hypothesis that genetic variation of IL-4 and IL-4Rα interact and play a central role in the regulation of pathogenic IgG4 antibody production or the clinical course of the disease. In contrast, polymorphisms of the *IL13* gene and *IL13RA2* (that encodes one IL-13 receptor chain; located in Xq24) were not associated with the disease (Toumi *et al.*, 2013).

Pemphigus has been considered a Th2 disease. However, support for the involvement of the IL23/Th17 pathway in the pathogenesis of pemphigus was found. In Tunisian PF, a higher frequency of circulating Th17 cells was observed in patients' blood compared to controls. Eleven tag SNPs in the IL23/Th17 axis genes *IL23R* (interleukin 23 receptor), *IL17A* (interleukin 17A), *IL17F* (interleukin 17F), *IL17RA* (interleukin 17 receptor A), *RORC* (RORγt), *TNF* (tumor necrosis factor) and *STAT3* (signal transducer and activator of transcription 3) genes were selected. The *IL23R rs11209026 G/G* genotype, the *IL17A rs3748067 C/C* genotype, the *IL17F rs763780 C* allele, and the *TNF −308G>A rs1800629 A* allele (in both the *A/A* and *A/G* genotypes) were associated with increased susceptibility (Ben Jmaa *et al.*, 2018).

The favored hypothesis about mechanisms underlying the associations of pemphigus with cytokine polymorphisms is that individuals with different genotypes for regulatory polymorphisms express different cytokine levels that may impact pathogenesis. The disease-associated *IL6*, *IL4, IL4R* and *TNF* SNPs cited above are eQTL (expression quantitative trait loci, which influence the transcription level of one or more genes) according to the GTEx Portal (Carithers *et al.*, 2015). Altered levels of cytokines were observed in the sera and lesional skin of pemphigus patients and possibly play a role in pathogenesis and disease severity (Ameglio *et al.*, 1999; Zeoti *et al.*, 2000; Timóteo *et al.*, 2017a,b; Ben Jmaa *et al.*, 2018). Moreover, the altered cytokine levels that occur in ADs are among the causes of the wide variation in responsiveness to glucocorticoid therapy. Augmented production of inflammatory cytokines may downregulate glucocorticoid receptor expression, resulting in diminished or lacking response to treatment (Yang *et al.*, 2012). Decreased glucocorticoid sensitivity associated with higher levels of IL-6 and TNFα was seen *in vitro* for PBMC from pemphigus patients (Chriguer *et al.*, 2012).

Genetic variants and the expression level of the BAFF cytokine has also been investigated in pemphigus and will be discussed in the following topic.

### The B lymphocyte co-stimulators CD40, CD40LG, BAFF, and CD19

A role for *CD40*, *CD40LG* and *BAFF* polymorphisms in pemphigus finds support in studies of protein or mRNA levels in PF and PV, and in the effects of these molecules and their genetic variants in homeostasis, in inflammation, and ADs.

CD40 (TNFRSF5) is a co-stimulatory molecule at the surface of a variety of cells like B lymphocytes, macrophages, and dendritic cells. In the skin, Langerhans cells and keratinocytes constitutively express CD40. Its ligand CD40LG (also known as CD40L TNFSF5, CD154, TRAP) is expressed at the surface of activated but not resting CD4+ T lymphocytes, and other hematopoietic and nonhematopoietic cells.

CD40/CD40LG interaction induces intracellular signals and expression of surface and secreted molecules required for antibody- and cell-mediated adaptive immune responses The CD40/CD40LG interactions also are essential for peripheral B lymphocyte tolerance. Lack of functional CD40LG or CD40 results in the monogenic immunodeficiency syndromes called hyper IgM syndrome type 1 (X-linked, OMIM #308230) and type 3 (autosomal recessive, OMIM #606843), respectively. Patients present normal or elevated serum IgM levels associated with markedly decreased IgG, IgA, and IgE, and reduced Treg frequency, as well as impaired immunoglobulin somatic hypermutation, class switch recombination, and repertoire selection. The patients are susceptible to recurrent or opportunistic infections and autoimmune manifestations.

The *CD40LG* gene is located at Xq26.3. The risk of FS is increased by homozygosity (in women) or hemizygosity (in men) for the major *T* allele of SNP *rs3092945* (-726T>C). No association was seen for *rs56074249* a 3' UTR(CA) short tandem repeat (STR, or microsatellite) (Malheiros and Petzl-Erler, 2009).

The *CD40LG rs3092945* SNP has not been considered as a marker in other studies of ADs, because it is absent or very rare all over the world, except for sub-Saharan African and some admixed populations of South and North America. However, associations with other polymorphisms of the *CD40LG* gene or close to it the were seen for ADs such as celiac disease, ulcerative colitis, and Crohn's disease (Li *et al.*, 2015).

The *CD40* gene is at the cytogenetic location 20q13.12. The 5' UTR polymorphism −1C>T (*rs1883832*) was analyzed in FS. This SNP resides in the Kozak sequence that includes the translation initiation codon (AUG) and the surrounding nucleotides and is important for ribosome binding to the mRNA. The *rs1883832 T* allele was significantly associated with decreased susceptibility to FS, consistent with a dominant or additive protective effect. Accordingly, the *C/C* genotype was associated with increased susceptibility to FS (Malheiros and Petzl-Erler, 2009).

Involvement of CD40/CD40LG levels was observed in the pathogenesis of pemphigus. Upregulation of both the receptor and the ligand has been reported in lesional skin and the serum of patients with active PV and PF. Numerous CD40LG+ cells and *CD40LG* mRNA copies were seen in lesional specimens compared to controls, and immunostaining for CD40 was intense both in the dermis and in keratinocytes. Additionally, patients' sera contained high levels of sCD40LG that is mainly secreted by activated T lymphocytes (Caproni *et al.*, 2007).

In FS patients, there is an increased number of dendritic cells in lesional skin, and this correlates with serum autoantibody titers (Chiossi *et al.*, 2004). It has been shown that the *CD40 rs1883832 C* allele increases the translational efficiency of nascent mRNA, resulting in 15% to 32% more CD40 protein than that seen for the *T* allele (Jacobson *et al.*, 2005). Altogether, these findings support the hypothesis that higher levels of CD40 in individuals with the *rs1883832 C* allele may contribute to the pathogenesis of FS.

Variable susceptibility to ATD, SLE and RA also is associated with *CD40* polymorphisms, especially in Europeans and specifically with the intron SNP *rs4810485* that is a proxy for *rs1883832* (r^2^ 1 in all analyzed non-African populations; 1000 genomes via LDlink) (Lee *et al.*, 2015a,b).

The *BAFF* (*TNFSF13B*) gene maps to 17p13.1. The B cell activating factor (BAFF, also known as BLYS, TNFSF13B, TALL1, THANK) is predominantly produced by myeloid cells, but regulated expression by many different hematopoietic and non-hematopoietic cell types has been described (Vincent *et al.*, 2013). BAFF is initially expressed as a membrane-bound trimer, which is proteolytically cleaved and released in a soluble form. Among its multiple effects, BAFF is a critical regulator of B lymphocyte differentiation, maturation, and survival. It is also involved in the immunoglobulin switch from IgM to IgG, IgE, and IgA. The homologous proliferation-inducing ligand (APRIL, TNFSF13A or TALL2) also has multiple effects in B lymphocyte biology; however, a possible impact of its genetic variants in pemphigus has not yet been published.

For FS, a weak protective association was found with the *T* allele of the *rs9514828* SNP (-871C>T, upstream of the *BAFF* gene transcription initiation site) (Malheiros and Petzl-Erler, 2009). This SNP is in the binding site of transcription factor MZF1 and may change its binding affinity, resulting in altered levels of BAFF (Kawasaki *et al.*, 2002). MZF1 was reported to be preferentially expressed in differentiating myeloid cells (Hromas *et al.*, 1991). In a genome-wide mRNA expression profile in FS, BAFF expression was significantly increased in CD4+ T lymphocytes of patients with active disease and decreased in patients under immunosuppressive treatment, both compared to healthy individuals, and also overexpressed in lesional skin compared to non-lesional skin of the same patients (Malheiros *et al.*, 2014). However, the 3' UTR SNPs *rs4145212*, *rs116898958,* and *rs185198828* that may alter the binding sites of microRNAs were not associated with FS (Cipolla *et al.*, 2016).

Remarkably, regarding susceptibility to FS, gene-gene interactions may occur between *BAFF* and both *CD40* and *CD40LG*. So, the protective effects of *CD40LG rs3092945 C* and *CD40 rs1883832 T* alleles only manifest in *BAFF rs9514828 T*-positive individuals, and vice versa (Malheiros and Petzl-Erler, 2009). This is not unexpected given the functional interactions between CD40, CD40LG, and BAFF in health and disease, and the effect of the genetic variants on protein levels. Notwithstanding, additional studies are needed to validate the associations and to understand their causes.

Functional effects of BAFF genetic variation have also been reported for SLE and MS in Sardinia (Steri *et al.*, 2017). Circulating BAFF levels are often elevated in patients with SLE and correlate with clinical disease activity. Elevated levels of BAFF were reported in the serum of RA and Sjögren's syndrome as well, but not PV (Asashima *et al.*, 2006).

The B lymphocyte antigen CD19 is expressed by early pre-B cells from the time of heavy chain rearrangement until plasma cell differentiation, and by follicular dendritic cells. Antigen-induced B cell receptor signaling is modulated by a multimolecular complex on the membrane of B lymphocytes of which CD19 functions as the principal component. CD19 is required for optimal antibody responses and selection against inherent autoreactivity. The autosomal recessive common variable immunodeficiency 3 (CVID3, OMIM #613493) is caused by lack of functional CD19. Among other alterations of B lymphocyte immunity, selection against the autoreactive properties of immunoglobulins is defective in patients (van Zelm *et al.*, 2014).

Recently, a unique CD19^hi^ B lymphocyte population exhibiting activation and memory-like properties was detected in the periphery of pemphigus patients. Genes involved in B lymphocyte activation and differentiation were up-regulated in these B cells. A tight correlation between peripheral CD19^hi^ B cells and total IgG/IgM levels was seen. These cells might contain B lymphocyte precursors for terminal differentiation and contribute to IgG/IgM production in ADs (Liu *et al.*, 2017).

These observations motivated the search for a possible association of FS with *CD19* variants. Two polymorphisms of the *CD19* gene (mapped to 16p11.2), intron SNP IVS14 −30C>T and an STR at the 3' UTR were used as markers. They had been previously associated with susceptibility to SLE in the Japanese population (Kuroki *et al.*, 2002). For pemphigus, no significant differences between the patient and control samples were seen such that these polymorphisms do not play any crucial role in the inter-individual variation of susceptibility (Malheiros and Petzl-Erler, 2009). Given these observations and the scarcity of studies, it would be premature to conclude that genetic variation of CD19 is irrelevant for pemphigus.

### Common genetic variants of the molecules involved in T lymphocyte activation and tolerance may influence susceptibility to pemphigus.

Adequate T lymphocyte response to antigen requires specific interaction of the peptide-HLA complex with the T cell receptor, as well as co-stimulatory and co-inhibitory signals that regulate activation, proliferation, and termination of the T cell response. The balance between positive and negative signals determines the outcome; hence, disruption of that balance may result in disease. These co-regulatory signals are provided by membrane-bound receptor-ligand pairs of which the most prominent are CD28/CTLA4:CD80/CD86, ICOS:ICOSL, and PD-1 (or PDCD1):PD-L1(CD274)/PD-L2, which are members of the immunoglobulin superfamily.

CD28-CD80/CD86 is the classical T lymphocytes costimulatory pathway. CTLA4 (or CD152) is an inhibitory receptor that can outcompete CD28, binding to CD80 and CD86 with higher affinity than CD28 and limiting T cell responses (Goronzy and Weyand, 2008). ICOS is an important co-stimulatory receptor, especially for Th2 effector cells. While CD28:CD80/CD86 interactions are critical for the initiation of an effective immune response, ICOS:ICOSL is required at later stages and predominates over CD28 for secondary immune responses (Coyle *et al.*, 2000). ICOS is critical for humoral immune responses. The co-inhibitory molecules PD-L1 and PD-L2 interact with the PD-1 receptor to suppress responses by T lymphocytes (Keir *et al.*, 2007).

Haploinsufficiency of, or impaired ligand binding to, CTLA4 result in a rare autosomal dominant immune dysregulation syndrome with incomplete penetrance named autoimmune lymphoproliferative syndrome type V (ALPS5 or CHAI; OMIM #616100). Common variable immunodeficiency 1 (CVID1; OMIM #607594) is an autosomal recessive disease due to mutations in *ICOS*.

For FS in Brazil, nineteen polymorphic markers were analyzed. For region 2q33.2, seven SNPs and three STR were selected, ranging from the promoter region of the *CD28* gene to the intergenic region between *CTLA4* and *ICOS*. A protective effect of allele *T* of *CTLA4 rs5742909* (-318C>T) was detected, while for *CTLA4 rs733618 (* −1722T>C) the *C* allele was associated with increased susceptibility. Another *CTLA4* SNP *rs139105990* in a putative microRNA binding site is not associated with FS (Cipolla *et al.*, 2016). For region 3q13.33, seven polymorphisms in the *CD80* promoter and one missense SNP of the *CD86* gene were analyzed. Significant associations occurred for *CTLA4* and *CD86* SNPs, and the STR (Dalla-Costa *et al.*, 2010).

The *rs5742909 T* allele marks higher promoter activity (Wang *et al.*, 2002) and increased expression of CTLA4 (Ligers *et al.*, 2001), which could lower the risk of ADs.

The *rs733618* risk allele *C* might lead to altered alternative splicing and decreased expression and function of membrane-bound CTLA4, resulting in impaired inhibition of T lymphocyte activation, which might contribute to the development of AD, as suggested for myastenia gravis (MG) (Wang *et al.*, 2008).

In the sample of predominantely African ancestry, lower risk of the disease was associated with allele *A* of the *CD86 rs1129055* (1057G>A, Ala304Thr) SNP, particularly when in homozygosis. This allele may alter the intracellular signal transduction pathways controlled by the CD86 molecule on antigen presenting cells; however, the lack of association in the Euro-Brazilian sample argues against a direct effect of the *rs1129055* polymorphism in susceptibility.

Three *PDCD1* polymorphisms were analyzed in FS: *rs11568821* (PD1.3, in intron 4), *rs2227981* (PD1.5, a synonymous SNP), *rs10204525* (PD1.6, a 3' UTR SNP). The *rs10204525* may influence binding of microRNA and transcription factors, altering the expression of PD-1. Its major allele *A* was over-represented among the patients of predominantly European ancestry, but not in the Afro-Brazilian sample (Braun-Prado and Petzl-Erler, 2007).

Analysis of a small sample of Polish PV (n = 40) and PF (n = 14) patients showed no statistically significant differences between the patients and the controls for the *CTLA4* missense polymorphism *rs231775* (+49A>G; Thr17Ala). For *ICOS*, carriers of the allele *C* of *rs10932029* (IVS1+173T>C) were more frequent between each of both patient samples in comparison with the control sample (Narbutt *et al.*, 2010).

### Genetics sheds light on the controversial relevance of the complement system in pemphigus

A primary message given by associations between complex diseases and (inherited) genetic polymorphisms is that the (mostly still unknown) mechanisms linking the genotype to disease susceptibility are causal in (rather than a consequence of) the pathogenic process.

The complement system (CS) consists of a large number of soluble and membrane-bound proteins and represents one of the major effector mechanisms of innate immunity against pathogens, and for removal of cellular debris and immune complexes. The role of complement in pemphigus has been a controversial issue, mainly because pathogenic anti-desmoglein autoantibodies mostly belong to the IgG4 subclass that does not initiate the classical complement pathway, and because acantholysis in pemphigus does not require complement *in vitro*. However, the alternative and the lectin complement pathways are not initiated by anti-gen/antibody complexes. Moreover, the CS is emerging as a global regulator of immune responses and tissue homeostasis, beyond its well-known role in innate immunity (Hajishengallis *et al.*, 2017, and others). Please refer to the recent article by Bumiller-Bini *et al.* (2018) for an appraisal of the debate about the role of complement in pemphigus.

Motivated by prior observations of altered expression of CD59 - the most essential MAC inhibitor - in several ADs, the hypothesis that SNPs in noncoding regions may regulate CD59 expression levels and participate in autoimmune pathogenic processes was tested in a recently published work (Salviano-Silva *et al.*, 2017). Six intronic and 3' UTR polymorphisms that might affect alternative splicing of the primary RNA transcript or regulation of mRNA stability in the cytoplasm were analyzed in a case-control FS association study, and for a possible effect on transcript levels. Specific alleles and haplotypes influenced disease susceptibility as well as mRNA expression levels, especially in women. The risk haplotype *G-G-C-C-A-A* also marked higher mRNA expression. The authors concluded that higher *CD59* transcriptional levels might increase susceptibility to FS (especially in women), possibly due to the role of CD59 in T lymphocyte signal transduction (Salviano-Silva *et al.*, 2017). Association with *rs1047581* was replicated in a subsequent study (Bumiller-Bini *et al.*, 2018, see below).

The complement receptor 1 (CR1, or CD35) plays a major role in inhibiting the complement system, removing immune complexes, and activating B cells. The gene contains several functional polymorphisms that have been associated with different multifactorial diseases (please refer to Oliveira *et al.*, 2019). In a study of FS, 11 *CR1* SNPs were analysed. Among these were the SNPs that define the Knops blood group system (York and McCoy antigens on erithrocytes). The haplotypes CR1*3B2B (York) and CR1*3A2A (with p.1208Arg) were associated with protection, and the CR1*1 haplotype (McCoy) with increased susceptibility. Furthermore, heterozygote *rs12034383 A/G* individuals presented higher CR1 mRNA levels than *G/G* homozygotes. The lowest soluble CR1 (sCR1) levels occurred in patients with active, more severe (generalized) disease, but treatment and remission resulted in the increase of median sCR1 levels. So, genetic variants of CR1 seem to modulate susceptibility to the disease and higher sCR1 levels may have an anti-inflammatory effect in patients with FS (Oliveira *et al.*, 2019).

A region in chromosome 9q33.2 that includes the complement component *C5* gene and the TNF receptor-associated factor 1 gene (*TRAF1*) had been identified as a susceptibility and severity factor for diverse diseases, such as RA and SLE (Kurreeman *et al.*, 2010). The SNP *rs10818488* is an eQTL for different genes and could have a functional impact on C5 synthesis. Even so, this regulatory intergenic SNP was not associated with PF and PV in the Tunisian population (Mejri *et al.*, 2009). In line with this result, *C5* polymorphisms were also not associated with FS (Bumiller-Bini *et al.*, 2018).

In a recent comprehensive study, 992 SNPs distributed within 44 CS genes were analyzed in a case-control study of FS (Bumiller-Bini *et al.*, 2018). Evidence for association was seen with variants of 10 genes that encode most of the complement proteins previously detected in the skin or presenting altered serum levels in patients (Table 1): *C3* (complement component 3), *C5AR1* (complement component 5a receptor 1, the primary receptor for C5a anaphylatoxin), *C8A* (complement component 8, alpha subunit, a component of the membrane attack complex MAC), *C9* (complement component 9 of the MAC), *CD59* (MAC inhibitor), *CFH* (complement factor H, the major regulator of the alternative pathway), *CR2* (complement receptor 2), *ITGAM* (integrin alpha-M or CR3, the alpha chain of a receptor for the iC3b fragment of C3), *ITGAX* (integrin alpha-X, or CR4, the alpha chain of a receptor for the iC3b fragment of the C3), and *MASP1* (mannan-binding lectin serine protease 1, an essential protein in the lectin pathway of complement) (Bumiller-Bini *et al.*, 2018).

### Epigenetic alterations of DNA and histones

In recent years, the involvement of epigenetic alterations in inflammatory and autoimmune diseases has been recognized and attracted much interest (Nielsen and Tost, 2013; Picascia *et al.*, 2015; Zhang and Lu, 2018). However, the molecular mechanisms underpinning these epigenetic changes in diseases are still poorly understood. Most studies on the effect of epigenetic mechanisms on complex diseases have been restricted to evaluation of the DNA methylation pattern.

For autoimmune skin diseases, it was observed that deregulation of DNA and histone methylation contributes to PV (Zhao *et al.*, 2012), psoriasis (Chandra *et al.*, 2015; O'Rielly *et al.*, 2015), systemic lupus erythematosus (SLE) (Hung *et al.*, 2018) among other. In PBMCs of patients with PV, genomic DNA methylation was increased relative to controls. DNA methyltransferase 1 (DNMT1) expression levels were significantly higher and methyl-CpG binding domain protein 3 (MBD3) levels were downregulated in patients compared with healthy controls. Global histone H3/H4 acetylation and H3K4/H3K27 methylation levels were significantly decreased in patient compared with healthy controls. These changes were accompanied by increased histone deacetylase 1 and 2 (HDAC1, HDAC2) and suppressor of variegation 3-9 homolog 2 (SUV39H2), and decreased SUV39H1 and enhancer of zeste 2 polycomb repressive complex 2 subunit (EZH2) in PV PBMCs (Zhao *et al.*, 2012).

For pemphigus, there are no published studies of variants in genes that act on epigenetic mechanisms, except for a recent study of FS (Spadoni *et al.*, 2020). A total of 566 polymorphisms in 63 genes that code for lysine methyltransferases (KMT), demethylases (KDM), DNA methyltransferases (DNMT) and ten-eleven translocation demethylases (TET) were considered in a case-control association study. Eleven SNPs in four genes were associated with FS: In the histone lysine demethylase 4C gene *KDM4C* (3 SNPs) and in the histone lysine methyltransferases genes *SETD7/KMT7* (1 SNP), *MECOM/KMT8E* (5 SNPs), and *PRDM16/KMT8F* (2 SNPs). The results of the study indicate that dysregulated histone (de)methylation plays a major role in pemphigus pathogenesis.

### Associations with variants of genes involved in regulated cell death pathways yield insight into the poorly understood cell death mechanism in pemphigus

Twelve regulated cell death (RCD) routes have been recognized: intrinsic apoptosis, extrinsic apoptosis, mitochondrial permeability transition (MPT)-driven necrosis, necroptosis, ferroptosis, pyroptosis, parthanatos, entotic, NETotic, lysosome-dependent, autophagy-dependent and immunogenic pathways (Galluzzi *et al.*, 2018). To date, only apoptosis has been considered in pemphigus, with controversial results about its role in the loss of cell adhesion and cell death. Some authors stated that cell death occurs by apoptosis (Gniadecki *et al.*, 1998; Rodrigues *et al.*, 2009), while others argued that there is no clear evidence of the occurrence of such an event in pemphigus (Lee *et al.*, 2009; Schmidt *et al.*, 2009; Janse *et al.*, 2014; Sokol *et al.*, 2015).

Frequencies of 1,167 SNPs from genes encoding products of all the 12 well-established cell death cascades were compared between FS patients and healthy control individuals (Bumiller-Bini *et al.*, 2019). Ten gene variants belonging to six cell death pathways differed significantly between these two population samples: necroptosis (*TNF* and *TRAF2*), apoptosis (*TNF, CD36* and *PAK2*), pyroptosis (*PRKN*), immunogenic cell death (*CD47, SIRPA* and *EIF2AK3*), parthanatos (*HK1*) and necrosis (*RAPGEF3*). The genetic association profile with *TNF, TRAF2, CD36* and *PAK2* variants marks decreased susceptibility to FS and higher TNF and TRAF2 levels and lover CD36 levels. This profile may favor cell survival and inflammation instead of apoptosis/necroptosis. Conversely, higher susceptibility is marked by variants of *CD47* and *SIRPA* of the immunogenic cell death pathway, proposed to lead to increased internalization of cell debris and antigen presentation, which may increment autoantibody production in FS.

### Receptors for the Fc portion of immunoglobulin G

Low-affinity Fcγ receptors bind the Fc portion of polymeric IgG in antigen-antibody immune complexes. They are cell-surface receptors expressed by different immune cells and mediate inflammatory responses. IgG binding can either activate or inhibit downstream cellular responses depending on the presence of ITAM or ITIM in the intracellular portion of the engaged Fcγ receptor. Dysregulation of Fcγ receptors is critical in diverse inflammatory and ADs (see Recke *et al.*, 2015).

Five closely linked paralogous genes at the cytogenetic location 1q23.3, *FCGR2A, FCGR2B, FCGR2C, FCGR3A,* and *FCGR3B,* encode the low-affinity receptors FcγRIIa, FcγRIIb, FcγRIIc, FcγRIIIa, and FcγRIIIb, respectively. Copy number variation occurs by deletion or duplication of *FCGR3A* and *FCGR2C* together, or *FCGR3B* and *FCGR2C* together, but not *FCGR2A* or *FCGR2B*. Common single-nucleotide variation within and between the paralogs adds another layer of complexity to the FCGR region.

Recke *et al.* (2015) estimated the effect of the patient *FCGR* genotype on the risk to develop PV/PF or BP (bullous pemphigoid) in a case-control study. The risk of PV/PF was decreased in the presence of allele C of the promoter polymorphism *rs3219018* (-386G>C) that affects the binding of transcription factors and expression levels of FcγRIIb (*FCGR2B*) and increased in the presence of an *FCGR2C rs183547105 ORF* allele. Because the inhibitory FcγRIIb is involved in peripheral tolerance of B lymphocytes, which may be counter-balanced by functional FcγRIIc expression, the authors proposed that these polymorphisms alter the risk of PV/PF due to a shift of the threshold for activation and proliferation of autoreactive B lymphocytes (Recke *et al.*, 2015).

### Does the variation of the forkhead box P3 gene *FOXP3* have any impact on pemphigus foliaceus?

The *FOXP3* gene is located at Xp11.23 and mutations in its coding region cause IPEX, the monogenic X-linked immune dysregulation, polyendocrinopathy, and enteropathy (OMIM #304790). Susceptibility to some multifactorial ADs has been associated with *FOXP3* polymorphisms (Oda *et al.*, 2013). *FOXP3* is a candidate for diseases with an immune background because it codes for a transcription factor of prime importance for the regulation of immune responses by T lymphocytes and in development of CD4+ CD25(IL2RA)+ Treg cells, which are critical for suppression of autoimmune or otherwise inappropriate immune responses. DSG3-induced Treg cells that inhibited autoreactive Th clones were preferentially isolated from the peripheral blood of healthy individuals who carried the PV-associated HLA class II alleles, HLA DRB1*04:02 and DQB1*05:03, and only from a minority of patients with PV. These results strongly suggest that these Treg cells may be involved in the maintenance of self tolerance against DSG3 (Veldman *et al.*, 2004).

Thus far, only in Tunisia PF has been analyzed in search of a possible influence of *FOXP3* variants in susceptibility. In a sample of women, the intronic SNPs *rs3761547* allele *G*, *rs3761548 A*, *rs3761549 C*, and *rs2294021 C* were associated with increased susceptibility to endemic PF. For sporadic PF, a weak association was seen only with *rs3761549 C* in the individual analysis, but higher susceptibility to both endemic and sporadic PF was associated with haplotype *G-A-15-C-C* where 15 stands for the allele of a (GT)_n_ STR in the promoter region (Ben Jmaa *et al.*, 2017). The genomic region of chromosome X that includes the *FOXP3* gene bears many protein-coding and noncoding RNA genes whose SNPs present very high LD (r^2^ ≥ 0.8) (1000 genomes via LDlink). The four SNPs analyzed in that study mark three different LD blocks. It would be relevant to verify if the association can be validated in other populations and for the various forms of pemphigus.

### The *ST18* gene

*ST18* encodes a transcription factor (zinc finger protein 387; ZNF387) and regulates apoptosis and inflammation, two processes relevant to pemphigus pathogenesis. *ST18* expression was upregulated in the skin of PV patients. The secretion of TNFα, IL-1a, and IL-6 that were reported to be increased in the lesioned skin of PV patients. In functional assays, these same cytokines were increased by *ST18* overexpression in the presence of PV serum or PV IgG. Allele A of the *ST18* polymorphisms *rs2304365* was associated with PV in Jews and Egyptians, but not in Germans (Sarig *et al.*, 2012). Subsequently, high LD between SNPs *rs2304365* and *rs17315309* was detected in Jews, and the authors concluded that the functional SNP *rs17315309* allele G, which drives *ST18* upregulation possibly is the causal polymorphism (Vodo *et al.*, 2016). The risk allele *rs2304365 A* was associated with severe pemphigus and a higher age of disease onset in the Iranian population (Etesami *et al.*, 2018). In the Chinese population, no association with *rs2304365* was seen for PV and PF (Yue *et al.*, 2014). The apparently conflicting results between the association studies in the Jewish and Chinese populations can be explained by the absence of the risk allele *rs17315309 G* in Eastern Asian populations (Ensembl, Cunningham *et al.*, 2019). However, the *rs17315309 G* allele is common, and LD with *rs2304365* is high in Europeans (LDlink), such that the lack of association with PV in the German population remains unexplained.

### Non-coding RNAs: The new players in complex phenotypes

A non-coding RNA (ncRNA) is a functional RNA molecule that is transcribed from DNA but not translated into a polypeptide. NcRNAs are involved in a wide range of biological processes, including gene transcription, post-transcriptional modifications, signal transduction, besides chromatin remodeling and other epigenetic mechanisms. Their deregulation or nucleotide sequence variation may contribute to disease.

For FS in Brazil, 2,080 SNPs located in long ncRNAs (lncRNAs) genes were evaluated in a case-control association study. Six of these polymorphisms possibly have an impact on susceptibility. The variant *rs7144332 T* in the lncRNA *AL110292.1* showed the most significant association with FS susceptibility. Results for five other lncRNA genes were suggestive of association*: rs6942557 C* in *LINC01176* and *rs17774133 T* in *LINC01119* were associated with increased risk; *rs6095016 A* in *lnc-PREX1-7:1*, *rs7195536 G* in *AC009121.1*, and *rs1542604 T* in *AC133785.1* were associated with decreased disease risk (Lobo-Alves *et al.*, 2019b). The function of these five lncRNA is elusive so far, but it was possible to suggest a functional impact of the SNPs on the lncRNAs structure, expression level, or interaction with microRNAs.

### Lack of association with some remarkable candidates

The lymphoid phosphatase LYP (also known as PTPN22 - protein tyrosine phosphatase, nonreceptor-type, 22) regulates signal transduction in immune cells. Among its many effects, LYP is a potent negative regulator of T lymphocyte activation (Hill *et al.*, 2002). Genetic variation of *PTPN22* (located at 1p13.2) is among the most influential genetic risk factors for ADs outside the MHC (Burn *et al.*, 2011). Increased risk has been associated with variants of *PTPN22*, notably with allele *T* of the missense *rs2476601* (1858C>T) single nucleotide polymorphism (SNP) that results in the Arg620Trp amino acid substitution in the first proline-rich motif of the LYP protein (Zheng *et al.*, 2012). The hypothesis that the *rs2476601 T* (620Trp) variant could be a shared risk variant for autoimmune and immune-mediated diseases has been raised (Smyth *et al.*, 2004).

Notwithstanding, for PF and PV, there is lack of association with *rs2476601*. The first study of PF and PV in the Tunisian population (Mejri *et al.*, 2007) provided evidence for no effect of the *PTPN22* variant on susceptibility; however, interpretation of the results was hampered because the frequency of allele *T* was low, impacting the statistical power of the analysis. Later on, in a North American population, the frequency of allele *T* was higher, of 7.8% in both the PV patient and control samples, and again no association was seen (Sachdev *et al.*, 2011). For FS, four SNPs (including *rs2476601*) in the genomic region 1p13.2, which together tag 28 SNPs on a segment of approximately 312,000 bp encompassing the *PTPN22*, *RSBN1*, *AL137856.1* genes and the 5' portion of the *AP4B1-AS1* gene, were used as markers. No significant association was found. Allele *rs2476601 T* was observed at the frequency of about 14% in both the patient and control samples (Lobo-Alves *et al.*, 2019a). So, variants in structural and regulatory sites of *PTPN22* and its flanking regions are not susceptibility factors for pemphigus, and it seems settled that the genetic variation of LYP has no impact on pemphigus disease susceptibility.

Most ADs not associated with *rs2476601* allele *T* (LYP 620Trp) manifest in skin, the gastrointestinal tract or in immune privileged sites, leading to the suggestion that the influence of that variant in susceptibility to ADs depends on the affected tissue, and that that the *PTPN22* polymorphism is not a shared susceptibility factor to antibody driven ADs (Zheng *et al.*, 2012). This opposition should be explored to deepen the understanding of the mechanisms that differentiate these two groups of ADs.

The *CD1D* gene encodes an MHC class I like glycoprotein (CD1d) whose primary function is presenting glycolipid antigens to natural killer T (NKT) cells. The *CD1D* mRNA is overexpressed in T CD4^+^ lymphocytes of FS patients when compared with healthy individuals (Malheiros *et al.*, 2014). The *CD1D* 3' UTR SNPs *rs16839951* and *rs422236* were not associated with the disease (Cipolla *et al.*, 2016). These results indicate that at least the analyzed genetic variants of CD1d do not contribute to FS susceptibility and that the reason for the previously observed expression difference between the samples of FS patients and unaffected individuals probably is a consequence of the pathogenic process. Also, the *KLRD1 rs2537752* and the *NKG7 rs3009* were not associated with FS (Cipolla *et al.*, 2016).

Cellular levels of various proapoptotic molecules, including Bax (BCL2-associated x protein) and p53 (tumor protein p53), are increased in pemphigus (Wang *et al.*, 2004). Because apoptosis dysregulation may play a role in pemphigus, genetic variants of the proteins involved in the apoptotic process may participate in the interindividual variation of susceptibility to the disease. Nonetheless, no effect on FS was observed for the *BAX* gene upstream regulatory region SNP *rs4645878* (-248G>A), nor for the missense *TP53 rs1042522* (12139C>G, Pro72Arg) SNP (Köhler and Petzl-Erler, 2006).

Associations reported for some cytokine genes were presented above, in a specific topic of this review. Genetic variants of some other cytokine and cytokine receptor genes were analyzed in PV and/or PF: *IL1A, IL1B, IL1R1, IL1RA, IL2, IL10, IL12, IL17RA, TNF, IFNG* (interferon gamma)*, LTA* (lymphotoxin alpha)*, MIF* (macrophage migration inhibitory factor) and *TGFB1* (transforming growth factor beta 1). No convincing associations were seen (Roxo *et al.*, 2003; Pereira *et al.*, 2004; Javor *et al.*, 2010; Saeedi *et al.*, 2013; Ben Jmaa *et al.*, 2018). However, for each of these genes, just one or a few selected SNPs were analyzed.

Lack of association with PF in Tunisia was also observed for variants of the transcription factors *RORC (RORGT)* and *STAT3* which stabilize and maintain Th17 cell function (Ben Jmaa *et al.*, 2018).

## Concluding remarks

Despite the successful identification of several genes and regulatory elements involved in pemphigus pathogenesis, knowledge in that field is still fragmentary, especially for PV. The consensus is that the HLA class II genes have the greatest effect in all forms of pemphigus and all populations. For several of the other genes, replication and validation studies are still lacking. Besides, many real associations certainly were overlooked, simply because the genes were not considered as candidates in published studies. It seemed that genome-wide association studies (GWAS) would permit pinpointing unsuspected non-coding and coding genetic elements. However, pemphigus are mostly rare, and unless very large patient (and control) samples are investigated, only highly significant associations with large effect sizes will be identified. This occurs because very stringent p-values are needed to avoid false-positives in GWAS. Consequently, many associations that may be relevant for the disease are missed in such studies. In fact, three recent GWAS of pemphigus in the Han Chinese population confirmed the great effect of HLA, but could not replicate weaker associations (Gao *et al.*, 2018, Sun *et al.*, 2019, Zhang *et al.*, 2019). Moreover, in both the GWAS and the hypothesis-driven candidate gene association studies, the effect of certain variants may remain undetected if specific epistatic interactions between two or more variants are missed because informative tag SNPs of the relevant variant(s) of the additional locus (or loci) are not available or were ignored. Improved analytical approaches for assessing evidence for associations between the disease and clusters of variants rather than just one or a few SNPs, may prove more informative in this regard.

Since the genotype is established before disease onset, it is the genotype that influences the disease, and not contrariwise. Thus, information on pemphigus genetics could support approaches to personalized medicine in the future. Nonetheless, observing an association with an SNP does not necessarily imply that the SNP is causal, because of linkage disequilibrium with one to many additional variants. This is a well-known phenomenon that emphasizes the need for fine-mapping and annotating the variants in the genomic region flanking the associated SNP in the particular study population to select the most likely causal SNP(s) for subsequent functional analyses. A critical next step will be to identify the effects of the putative risk variant(s) to understand the disease mechanisms. To achieve this, genetic engineering approaches, such as CRISPR technology-based genome editing, as well as novel techniques to detect DNA-DNA, DNA-RNA, RNA-RNA interactions, and DNA- or RNA-protein interactions combined with the information by expression quantitative trait loci (eQTL) studies will provide insight into the functional impact of non-coding variants on altered cellular phenotypes.

Epigenetic modifications such as DNA methylation and histone modification whose dysregulation can also be implicated in tolerance breakdown and pathogenic autoimmunity add another layer of complexity. Various epigenetic modifications are sensitive to external stimuli and may bridge the gap between the genome and the environment. Therefore, besides mapping epigenetic modifications in health and disease, a better appraisal of the relevant environmental factors is needed. Additionally, key cell types and cell states that may be implicated in pemphigus pathogenesis should be defined. Functional genomic annotations from these cell types and states can then be used to determine candidate genes and regulatory sequences, and the causal variants. Together with longitudinal studies, these approaches may produce crucial insights into how pemphigus develops. The growing understanding of the genetics and epigenetics of autoimmune disease may facilitate early diagnosis, refine the disease phenotypes, and improve therapeutic intervention.

## Supplementary material

The following online material is available for this article:

Table S1 - HLA class II associations for pemphigus vulgaris.

Table S2 - HLA class II associations for pemphigus foliaceus.
