# Supplementary material for: Beyond the HLA polymorphism: a complex pattern of genetic susceptibility to pemphigus
Source: Genet Mol Biol. 2020 Jul 1;43(3):e20190369. doi: 10.1590/1678-4685-GMB-2019-0369 (PMC7341728; doi:10.1590/1678-4685-GMB-2019-0369)
Supplement: Supplementary file 1 [file 1415-4757-GMB-43-3-e20190369-s1.pdf]

Supplementary Material to "Beyond the HLA polymorphism: a complex pattern of genetic susceptibility to pemphigus"

Table S1 - HLA class II associations described for pemphigus vulgaris in different populations.

Gene	Susceptibility	OR or RR	P value	Protective	OR or RR	P value	Comment	HLA typing method	Population	Sample size (individuals)	Reference
HLA-DRB1 allele frequency	DR4(DRB1*04)	4.02(1.99-8.10)	<0.0001	none			Only HLA-DR allele groups were analyzed. In parenthesis: actual nomenclature of DR and DQ allele groups. OR were calculated on basis of the data presented in the article	microlymphocytotoxicity; RFLP for DR4 and DQ subtyping	Ashkenazi Jews in USA	P: 26; C: 59	Ahmed AR, Yunis EJ, Khatri K, Wagner R, Notani G, Awdeh Z, Alper CA. Major histocompatibility complex haplotype studies in Ashkenazi Jewish patients with pemphigus vulgaris. Proc Natl Acad Sci U S A. 1990 Oct;87(19):7658-62.
HLA-DQB1	DQw8(DQB1*03:02)	na	na	na							
HLA-B_BF_C2_C4A_C4B_HLA-DR haplotype description	B38_S_C_2_1_DR4, B35_S_C_3_1_DR4	na	0.012, 0.011	na			BF_C2_C4A_C4B alleles form the "complotype", ie, the haplotype that includes alleles of these 4 complement components loci. Complotypes were not associated in the absence of HLA-DR4				
HLA-DRB1 individuals with the allele	DRB1*04(04:02), 08(08:04), 14(14:01#)	3.18(1.93-5.24), 2.97(1.63-5.42), 3.50(1.83-6.69)	6x10-6, 4x10-4, 2x10-4	DRB1*01, 03, 13, 15	0.18(0.04-0.73), 0.33(0.12-0.92), 0.26(0.09-0.72), 0.27(0.08-0.86)	0.016, 0.034, 0.010, 0.027	High resolution (4-digit) typing of groups DRB1*04, 08 and 14: 28/29 of 04 P were 04:02, 1 was 04:06; 15/18 of 08 P were 08:04; 14/16 of 14 P were 14:01#, 1 was 14:02 and one 14:04	PCR-SSP	Brazilians (admixed of predominantly European ancestry)	P: 51, C: 297	Gil JM, Weber R, Rosales CB, Rodrigues H, Sennes LU, Kalil J, Chagury A, Miziara ID. Study of the association between human leukocyte antigens (HLA) and pemphigus vulgaris in Brazilian patients. Int J Dermatol. 2017 May;56(5):557-562.
HLA-DQB1 individuals with the allele	DQB1*03:02, 05:03	4.14(2.47-6.94), 3.99(1.93-8.24)	1x10-5, 0.002	DQB1*02, 05:01, 06	0.33(0.16-0.67), 0.39(0.16-0.91), 0.56(0.31-1.02)	0.0024, 0.029, 0.034	OR and P for groups DQB1*02 and 06 calculated on basis of the data presented in the article				
HLA-DRB1-DQA1-DQB1 haplotype frequency	04-03:01-03:02, 14-01-05:03	4.73(2.76-8.12), 3.64(1.73-7.65)	2x10-8, 7x10-4	ni							
HLA-DRB1 allele frequency	DRB1*04, 04:02, 08:04, 14, 14:01, 14:04	24.88(16.59-37.33), 12.54(8.79-17.88), 6.0(3.29-10.97), 6.68(4.39-10.10), 7.21(4.72-10.99), 16.64(5.65-48.95)	p<0.0001, 5x10-10, 5x10-5, <0.0001, 5.4x10-10, 0.0005	DRB1*07:01, 11, 13	0.28(0.12-0.67), 0.50(0.29-0.87), 0.32(0.16-0.62)	0.027, 0.0124, 0.00047	OR and P for groups DRB1*04, 11, 13 and 14 calculated on basis of the data presented in the article	PCR-SSP	Brazilians (admixed of predominantly European ancestry)	P: 83; C: 1592	Brochado MJF, Nascimento DF, Deghaide NHS, Donadi EA, Roselino AM. Data on HLA class I/II profile in Brazilian pemphigus patients. Data Brief. 2016 Jun 3;8:364-74.
HLA-DQA1 allele frequency	DQA1*03, 03:01	2.83(2.02-3.97), 3.64(2.29-5.75)	<0.0001, 0.0004	DQA1*02:01, 05	0.34(0.15-0.76), 0.64(0.44-0.96)	0.0065, 0.0028	OR and P for groups DQA1*03, 05 and allele 02:01 calculated on basis of the data presented in the article				
HLA-DQB1 allele frequency	DQB1*03, 03:02, 05, 05:03	1.96(1.43-2.68), 2.95(2.30-3.80), 1.86(1.30-2.65). 2.74(1.49-5.08)	<0.0001, 3x10-10, 0.0005, 0.02	DQB1*02, 06, 06:02	0.27(0.14-0.54), 0.29(0.16-0.51), 0.19(0.06-0.60)	<0.0001, <0.0001, 0.0075	OR and P for groups DQB1*02, 03, 05 and 06 calculated on basis of the data presented in the article				

Gene	Susceptibility	OR or RR	P value	Protective	OR or RR	P value	Comment	HLA typing method	Population	Sample size (individuals)	Reference
HLA-DRB1-DQA1-DQB1 haplotype frequency	04-03-03, 14-01-05, 08-05-03	2.52(1.98-3.19), 7.87(5.37-11.5), 8.85(3.40-22.99)	2x10-10, 2x10-17, 0.0002	15-01-06, 13-01-06, 07-02-02, 03-05-02	0.27(0.10-0.72), 0.33(0.16-0.69), 0.35(0.14-0.83), 0.35(0.16-0.93)	0.0059, 0.002, 0.016, 0.036					Brochado MJ, Nascimento DF, Campos W, Deghaide NH, Donadi EA, Roselino AM. Differential HLA class I and class II associations in pemphigus foliaceus and pemphigus vulgaris patients from a prevalent Southeastem Brazilian region. J Autoimmun. 2016 Aug;72:19-24.
HLA-DRB1 individuals with the allele	DRB1*04, 04:02, 14, 14:01#, 08	3.21(1.66-6.22), 18.23(6.12-56.78), 5.07(2.24-11.52), 12.86(4.47-38.17), 2.49(1.11-5.53)	0.0003, 10-8, 0.00002, 10-6, 0.024	DRB1*03, 15	0.25(0.06-0.91), 0.09(0.00-0.63)	0.032, 0.007	Frequencies of the non-associated DRB1 and DQB1 alleles and allele groups were not informed	PCR-SSO	Argentinian (predomunantely European ancestry)	P: 47; C: 199	Glorio R, Rodriguez Costa G, Haas R, Gruber M, Fainboim L, Woscoff A. HLA haplotypes and class II molecular alleles in Argentinian patients with pemphigus vulgaris. J Cutan Med Surg. 2002 Sep-Oct;6(5):422-6.
HLA-DQB1 individuals with the allele	DQB1*03:02, 05:03	11.43(5.07-26.02), 8.02(3.03-21.48)	< 0.0001	ni							
HLA-DRB1 allele frequencies	DRB1*04, 04:02, 14, 14:01#	3.25, 40.09, 2.74, 47.6	0.0008, 3x10-13, 0.015, 0.00002	DRB1*03	0.30	0.041	95% CI not informed. Authors comment that DQB1*03:02 was associated because of LD with DRB1*04:02: no longer associated in the absence of DRB1*04:02	PCR-SSO	Venezuelan, admixed	P:49; C: 101	Sáenz-Cantele AM, Fernández-Mestre M, Montagnani S, Calebotta A, Balbas O, Layrisse Z. HLA-DRB1*0402 haplotypes without DQB1*0302 in Venezuelan patients with pemphigus vulgaris. Tissue Antigens. 2007 Apr;69(4):318-25.
HLA-DQB1 allele frequencies	DQB1*05:03	3.4	0.023	DQB1*03:01	0.25	0.00087					
HLA-DRB1 allele frequencies	DRB1*04:02, 14:01#, 14:04	34.03(12.2-89.40), 10.10(3.50-29.30), 204.02(9.2-5864.1)	2x10-14, 2x10-6, 9x10-6	none			Frequencies of the non-associated DRB1, DQA1 and DQB1 alleles and allele groups were not informed. The authors comment that DQA1*05:05 might have been misclassified as 05:01 in some studies	PCR-SSP	Non-Jewish USA	P: 26; C: 1899	Lee E, Lendas KA, Chow S, Pirani Y, Gordon D, Dionisio R, Nguyen D, Spizuoco A, Fotino M, Zhang Y, Sinha AA. Disease relevant HLA class II alleles isolated by genotypic, haplotypic, and sequence analysis in North American Caucasians with pemphigus vulgaris. Hum Immunol. 2006 Jan-Feb;67(1-2):125-39.
HLA-DQA1 allele frequencies	DQA1* 01:04, 03:01, 05:05	11.83(4,7-30,0), 4.36(1.9-10.2), 19.13(28.3-11409.3)	2.3x10-9, 9.0x10-6, 5.4x10-14	DQA1*05:01	0.45(0.004-1.0)	1.3x10-6					
HLA-DQB1 allele frequencies	DQB1*03:02, 05:03	3.77(1.6-8.8), 14.12(5.4-37.1)	6.8x10-5, 1.4x10-9	none							
HLA-DRB1 allele frequencies	DRB1*04, 04:02, 14, 14:04, 14:54	3.46(2.06-5.83), na, 7.14(3.11-10.96), 9.89(1.27-210.33), 4.80(2.05-11.65)	<0.00001, <0.00001, <0.00001, 0.0009, 0.00007	DRB1*03:01, 07:01, 15	0.26(0.10-0.61), 0.46(0.22-0.95), 0.30(0.12-0.73)	0.0009, 0.034, 0.005	Frequencies of DRB1 and DQB1 alleles and allele groups not associated in IndoAsians, nor in European in London were not informed	PCR-SSP	European / London	P: 96; C: 100	Saha M, Harman K, Mortimer NJ, Binda V, Black MM, Kondeatis E, Vaughan R, Groves RW. Pemphigus vulgaris in White Europeans is linked with HLA Class II allele HLA DRB1*1454 but not DRB1*1401. J Invest Dermatol. 2010 Jan;130(1):311-4.
HLA-DQB1 allele frequencies	DQB1*03:02, 05:03	4.94(2.70-9.11), 6.93(3.03-16.48)	<0.00001, <0.00001	DQB1*02, 03:03, 05:01, 06:02	0.32(0.17-0.59), 0.23(0.05-0.87), 0.41(0.19-0.87), 0.25(0.09-0.66)	0.0001, 0.027, 0.018, 0.0029					

Gene	Susceptibility	OR or RR	P value	Protective	OR or RR	P value	Comment	HLA typing method	Population	Sample size (individuals)	Reference
HLA-DRB1 allele frequencies	DRB1*04, 04:02, 14, 14:54+14:01	2.88(1,59-5.23), 112.2(6.73-1870.45), 13.43(3.86-46.67), 8.68(2.42-31.11)	0.00048, 4X10-12, 7x10-7, 0.00016	DRB1*01, 03, 13, 04:01:01	0.10(0.01-0.75), 0.27(0.09-0.83), 0.22(0.08-0.67), 0.10(0.01-0.75)	0.0062, 0.0164, 0.0038, 0.0062	OR and P calculated on basis of the data presented in the article. Only for groups DRB1*04, 08 and 14 high resolution typing was informed.	PCR-SSP for low resolution, SBT for 4-digit alleles	German	P: 46; C: 74	Haase O, Alneebari R, Eldarouti MA, Abd El Hady M, Dorgham D, El-Nabarawy E, El Din Mahmoud SB, Mosaad El Sayed H, Darwish M, Abbas F, Salah S, Mosaad Y, El-Chennawi F, Al Mongy S, Abdelaziz AM, Abd El Gaber S, Hertl M, Eming R, Recke A, Möller S, Schmidt E, Zillikens D, Ibrahim S. Association with HLA-DRB1 in Egyptian and German pemphigus vulgaris patients. Tissue Antigens. 2015 Apr;85(4):283-6.
HLA-DQB1 allele frequencies	DRB1*04:02, 14:01#, 14:04	91.6, 3.1, 17.2	5x10-16, 0.01, 4x10-5	none			95% CI not informed. Frequencies of the non-associated DRB1 and DQB1 alleles and allele groups were not informed	PCR-SSO and PCR-SSP/RFLP	French	P: 37; C: 106	Loiseau P, Lecleach L, Prost C, Lepage V, Busson M, Bastuji-Garin S, Roujeau JC, Charron D. HLA class II polymorphism contributes to specify desmoglein derived peptides in pemphigus vulgaris and pemphigus foliaceus. J Autoimmun. 2000 Aug;15(1):67-73.
HLA-DQB1 allele frequencies	DQB1*03:02, 05:03	6.9, 5.3	10-6, 5x10-5	DQB1*02	0.16	7x10-4					
HLA-DRB1 allele frequencies	DRB1*04, 04:02, 04:04, 14, 14:04, 14:05, 14:54	5.79(3.09-10.82), 22.75(7.62-67.86), 30.57(1.67-559.40), 10.40(3.99-27.10), 24.71(1.32-464.28), 18.99(0.97-371.80), 7.17(2.44-21.03)	<0.0001, <0.0001, 0.0015, <0.0001, 0.0055, 0.0204, 0.0002	DRB1*07, 13, 15	0.29(0.10-0.83), 0.27(0.09-0.77), 0.16(0.04-0.70)	0.02, 0.01, 0.005	Allele DRB1*14:54 was identified, corroborating that associations reported before with 14:01 probably are 14:54#	PCR-SSP	Slovak	P: 43; C: 113	Párnická Z, Švecová D, Javor J, Shawkatová I, Buc M. High susceptibility to pemphigus vulgaris due to HLA-DRB1*14:54 in the Slovak population. Int J Immunogenet. 2013 Dec;40(6):471-5.
HLA-DQB1 allele frequencies	DQB1*03:02, 05:03	7.70(3.82-15.54), 10.40(3.99-27.10)	<0.0001, <0.0001	DQB1*05:02, 06	0.14(0.02-1.10), 0.12(0.03-0.38)	0.03, <0.0001	Susceptibility groups: DQB1*03 and 05. DRB1*02 not associated. Association with DQB1*05:02 may result from linkage disequilibrium: It is associated with decreased risk in Slovak and increased risk in Iranian and Japanese.				
HLA-DRB1-DQB1 haplotype frequencies	14-05:03, 14:54-05:03, 14:04-05:03, 04-03:02, 04:02-03:02, 04:04-03:02	9.71(3.70-25.43), 7.17(2.44-21.03), 24.71(1.32-464.28), 7.70(3.82-15.54), 22.75(7.62-87.86), 24.71(1.34-464.28)	<0.0001, 0.0002, 0.0055, <0.0001, <0.0001, 0.0055	none							
HLA-DRB1 individuals with the allele	DRB1*04, 04:02, 14, 14:01#, 14:04, 08 (Identified as 08:04 by *Carcassi et al. 1996)	2.84(1.39-5.78), 15.61(5.04-48.31), 11.57(5.54-24.16). 5.98(2.86-12.52), 24.90(3.11-199.56), 4.68(1.35-16.21)	0.0046, 5x10-8, 5x10-12, 1x10-6, 6x10-5, 0.028, 0.013	DRB1*07 (03:01 also protective: *Carcassi et al. 1996)	0.19(0.06-0.55)	0.00087	OR and P calculated on basis of the data presented in the article. Only for groups DRB1*04 and 14 and for DQB1 high resolution typing was informed.	PCR-SSP	Italian (South)	P: 61; C: 128	Lombardi ML, Mercuro O, Ruocco V, Lo Schiavo A, Lombari V, Guerrera V, Pirozzi G, Manzo C. Common human leukocyte antigen alleles in pemphigus vulgaris and pemphigus foliaceus Italian patients. J Invest Dermatol. 1999 Jul;113(1):107-10.

Gene	Susceptibility	OR or RR	P value	Protective	OR or RR	P value	Comment	HLA typing method	Population	Sample size (individuals)	Reference
HLA-DQB1 individuals with the allele	DQB1*03:02, 05:03, 04:02	4.28(1.99-9.20), 17.57(7.85-39.36), 5.63(1.06-29.87)	0.0002, 2x10-14, 0.037	DQB1*02 (identified as 02:01 by *Carcassi et al. 1996), 06	0.35(0.16-0.77), 0.44(0.20-0.99)	0.0084, 0.044					*Carcassi C, Cottoni F, Floris L, Vacca A, Mulargia M, Arras M, Boero R, La Nasa G, Ledda A, Pizzati A, Cerimele D, Contu L. HLA haplotypes and class II molecular alleles in Sardinian and Italian patients with pemphigus vulgaris. Tissue Antigens. 1996 Dec;48(6):662-7.
HLA-DRB1 allele frequencies	DRB1*04, 04:02, 14, 14:04, 14:54	4.75(3.29-6.86), 15.47(6.38-37.52), 7.68(5.25-11.23), na, 2.69(1.26-5.72)	5x10-14, 7×10−14, 4x10-20, 2x10-8, 0.008	DRB1*01, 01:01, 03, 03:01, 11, 13, 13:02, 16, 16:01	0.45(0.20-0.96), 0.22(0.06-0.74), 0.36(0.16-0.81), 0.35(0.14-0.87), 0.37(0.19-0.70), 0.49(0.26-0.94), 0.09(0.01-0.69), 0.48(0.23-0.99), 0.36(0.14-0.91)	0.033, 0.008, 0.008, 0.019, 0.0008, 0.031, 0.004, 0.039, 0.025		PCR-SSP	Serbian	P: 72; C: DRB1 allele groups 1992, four-digit DRB1 112	Zivanovic D, Bojic S, Medenica L, Andric Z, Popadic D. Human leukocyte antigen class II (DRB1 and DQB1) alleles and haplotypes frequencies in patients with pemphigus vulgaris among the Serbian population. HLA. 2016 May;87(5):367-74. doi: 10.1111/tan.12796. Epub 2016 Apr 12. PubMed PMID: 27072514.
HLA-DQB1 allele frequencies	DQB1*03:02, 05:03	4.31(2.46-7.56), 3.87(2.13-7.04)	9.9x10-8, 3.7x10-6	DQB1*02, 02:01, 03:01, 06:04	0.44(0.23-0.86), 0.35(0.14-0.87), 0.42(0.21-0.85), 0.20(0.04-0.87)	0.014, 0.019, 0.014, 0.018					
HLA-DRB1-DQB1 haplotype frequencies	04-03, 04:02-03:02, 14-05, 14:04-05:03, 14:54-05:03	4.41(2.60-7.47), 15.0(6.19-36.61), 6.14(3.44-10.94), na, 2.50(1.12-5.55)	6.4x10-9, 1.9x10-13, 2.5x10-11, 1.3x10-7, 0.021	01-05, 03-02, 03:01-02:01, 11-03, 13:02-06:04, 16-05, 16:01-05:02	0.35(0.15-0.79), 0.29(0.12-0.71), 0.36(0.14-0.89), 0.37(0.18-0.74), 0.10(0.01-0.77), 0.37(0.16-0.84), 0.39(0.16-0.99)	0.009, 0.004, 0.022, 0.041, 0.007, 0.014, 0.041					
HLA-DRB1 allele frequencies	DRB1*04:02	4.96(2.20-11.4)	2x10-7	DRB1*03:01, 11:04, 07	0.05(0.01-1.60), 0.16(0.03--0.90), 0.15(0.03-0.80)	5x10-4, 4x10-4, 2x10-4	Frequencies of the non-associated DRB1, DQA1 and DQB1 alleles and allele groups were not informed. The authors comment that DQA1*05:05 might have been misclassified as 05:01 in some studies.	PCR-SSP	Ashkenazi Jews USA	P: 32; C: 132	Lee E, Lendas KA, Chow S, Pirani Y, Gordon D, Dionisio R, Nguyen D, Spizuoco A, Fotino M, Zhang Y, Sinha AA. Disease relevant HLA class II alleles isolated by genotypic, haplotypic, and sequence analysis in North American Caucasians with pemphigus vulgaris. Hum Immunol. 2006 Jan-Feb;67(1-2):125-39.
HLA-DQA1 allele frequencies	DQA1*03, 05:05	3.22(1.30-6.70), 97.33(0.02-25094782.)	6x10-5, 2x10-7	DQA1*01:01, 01:02, 02:01, 05:01	0.24(0.07-0.8), 0.08(0.004-1.2), 0.17(0.03-0.90), 0.01(0.002-0.30)	1x10-6, 3x10-4, 4x10-4, 2x10-16					
HLA-DQB1 allele frequencies	DQB1*03:02	3.0(1.4-6.8)	0.00013	DQB1*02:02/02:01, 03:01	0.02(0.003-0.5), 0.19(0.06-0.6)	2x10-10, 5x10-6					

Gene	Susceptibility	OR or RR	P value	Protective	OR or RR	P value	Comment	HLA typing method	Population	Sample size (individuals)	Reference
HLA-DRB1 allele frequencies	DRB1*04, 04:02, 08, 08:04, 14, 14:54+14:01	3.56(1.99-6.38), 9.03(4.08-19.97), 11.56(2.54-52.49), 17.26(2.17-137.23), 12.6(2.79-56.84), 25.11(1.42-445.15)	0.00002, 2x10-9, 0.00014, 0.00046, 0.00006, 0.0012	DRB1*11, 13, 15	0.23(0.07-0.82), 0.30(0.13-0.67), 0.17(0.04-0.73)	0.018, 0.0030, 0.012	OR and P calculated on basis of the data presented in the article. Only for groups DRB1*04, 08 and 14 high resolution typing was informed.	PCR-SSP for low resolution, SBT for 4-digit alleles	Egyptian	P: 47; C: 73	Haase O, Alneebari R, Eldarouti MA, Abd El Hady M, Dorgham D, El-Nabarawy E, El Din Mahmoud SB, Mosaad El Sayed H, Darwish M, Abbas F, Salah S, Mosaad Y, El-Chennawi F, Al Mongy S, Abdelaziz AM, Abd El Gaber S, Hertl M, Eming R, Recke A, Möller S, Schmidt E, Zillikens D, Ibrahim S. Association with HLA-DRB1 in Egyptian and German pemphigus vulgaris patients. Tissue Antigens. 2015 Apr;85(4):283-6.
HLA-DRB1 allele frequencies	DRB1*04, 04:02, 14	5.7, 13.9, 10.0	8x10-13, 0.002, 1x10-20	DRB1*03, 04:03, 11, 13, 15	0.10, 0.03, 0.25, 0.20, 0.40	0.0009, 2x10-8, 8x10-8, 0.001, 0.04	95% CI not informed. Individuals with two susceptibility alleles are at higher risk. Genotypes with a susceptibility and a protective allele (04/11) are neutral	PCR-SSP	Syrian	P: 91; C: 270	Harfouch E, Daoud S. Allelic variation in HLA-DRB1* loci in Syrian pemphigus vulgaris patients. Int J Dermatol. 2014 Dec;53(12):1460-3.
HLA-DRB1 allele frequencies	DRB1*04, 04:02, 14:01#	3.20(2.30-4.30), 7.16(3.29-15.77) , 3.18(2.30-4.30)	<0.0001, <0.0001, 0.0001	DRB1*15, 03:01, 07, 11	0.32(0.12-0.84), 0.24(0.06-0.94), 0.11(0.03-0.4), 0.54(0.32-0.91)	0.007, 0.01, <0.0001, 0.01		PCR-SSP	Iranian	P: 52; C: 180	Shams S, Amirzargar AA, Yousefi M, Rezaei N, Solgi G, Khosravi F, Ansaripour B, Moradi B, Nikbin B. HLA class II (DRB, DQA1 and DQB1) allele and haplotype frequencies in the patients with pemphigus vulgaris. J Clin Immunol. 2009 Mar;29(2):175-9.
HLA-DQA1 allele frequencies	DQA1*01:04, 03:01:01	2.44(1.50-3.20), 1.50(1.13-2.04)	<0.0001, 0.008	DQA1*01:01, 01:03, 02:01, 05	0.20(0.05-0.80), 0.43(0.18-1.02), 0.21(0.05-0.80), 0.47(0.30-0.74)	0.006, 0.04, 0.007, 0.0005					
HLA-DQB1 allele frequencies	DQB1*03:02, 03:05, 05:02, 06:03	3.85(2.80-5.10), 3.00(1.60-5.40), 3.50(2.50-4.70), 3.85(2.50-5.70)	<0.0001, 0.02, <0.0001, 0.002	DQB1*02:01, 03:01, 06:01:01, 06:02	0.16(0.05-0.50), 0.47(0.29-0.77), 0.16(0.02-1.12), 0.20(0.05-0.80)	<0.0001, 0.001, 0.01, 0.004					
HLA-DRB1-DQA1-DQB1 haplotype frequencies	04-03:01:01-03:02, 14:01#-01:04-05:02	3.95(2.95-5.28), 3.34(2.45-4.56)	<0.0001, <0.0001	03:01-05:01:01-02:01, 07-02:01-02:01, 11-05-03:01:01,15-01:03-06:01:01	0.29(0.06-0.96), 0.26(0.06-1.02), 0.60(0.36-1.01), 0.17(0.02-1.17)	0.01, 0.02, 0.05, 0.02					
HLA-DRB1 allele frequencies	DRB1*04, 04:02, 14, 14:04	4.01(1,54-10.8), 19.10(2.59-39.30), 67.45(3.16-13.35), 10.0(4.20-22.50)	0.002, 0.0003, <0.00001, <0.00001	DRB1*03:01, 15	0.05(0.0-0.38), 0.17(0.005-0.49)	0.0003, 0.0003	Frequencies of DRB1 and DQB1 alleles and allele groups not associated in IndoAsians, nor in European in London were not informed	PCR-SSP	Indo-Asians / London	P: 57; C: 59	Saha M, Harman K, Mortimer NJ, Binda V, Black MM, Kondeatis E, Vaughan R, Groves RW. Pemphigus vulgaris in White Europeans is linked with HLA Class II allele HLA DRB1*1454 but not DRB1*1401. J Invest Dermatol. 2010 Jan;130(1):311-4.
HLA-DQB1 allele frequencies	DQB1*03:02, 05:03	2.58(1.00-6.80), 6.18(3.07-12.57)	0.049, <0.00001	DQB1*02, 06:01	0.19(0.07-0.49), 0.25(0.08-0.75)	0.0001, 0.011					

Gene	Susceptibility	OR or RR	P value	Protective	OR or RR	P value	Comment	HLA typing method	Population	Sample size (individuals)	Reference
HLA-DRB1 frequency of individuals with allele	DRB1*04:06, 14:01#, 14:05	9.80(3.52-27.31), 11.10(3.55-34.68), 9.19(2.71-31.16)	0.00011, 0.00048, 0.0029	ni			OR and P for DRB1, DQA1, DQB1 and the haplotypes calculated on basis of the data presented in the article. Frequencies of protective and not associated DRB1, DQA1 and DQB1 alleles and allele groups were not informed. Association with DQB1*05:02 may result from linkage disequilibrium: It is associated with decreased risk in Slovak and increased risk in Iranian and Japanese.	PCR-RFLP	Japanese	P: 17; C: 525	Yamashina Y, Miyagawa S, Kawatsu T, Iida T, Higashimine I, Shirai T, Kaneshige T. Polymorphisms of HLA class II genes in Japanese patients with pemphigus vulgaris. Tissue Antigens. 1998 Jul;52(1):74-7.
HLA-DQA1 frequency of individuals with allele	DQA1*01:04, 03:01	14.42(5.26-39.51), 6.26(2.34-16.70)	0.000002, 0.00047	ni							
HLA-DQB1 frequency of individuals with allele	DQB1*05:02, 05:03:01, 03:02	10.01(2.51-39.91), 10.91(3.73-31.89), 5.44(2.04-14.47)	0.0076, 0.00017, 0.0011	ni							
HLA-DRB1-DQA1-DQB1 frequency of individuals with haplotype	04:06-03:01-03:02, 14:01#-01:04-05:02, 14:01# or14:05-01:04-05:03:01	9.80(3.52-27.31), 10.01(2.51-39.91), 10.91(3.73-31.89)	0.00011, 0.0076, 0.00017	ni							
HLA-DRB1	DRB1*14, 14:04, 14:05, 14:54	35.47, 61.86, 9.47, 2.70	4.2x10-63, 1.3x10-34, 1.4x10-23, 3.1x10-4	ni			95% CI not informed. Frequencies of DRB1 and DQB1 alleles and allele groups not associated were not informed. HLA genotypes were imputed from GWAS data and validated by NGS.	GWAS (microarray) and NGS-based HLA typing	Chinese (Han)	P: 210; C: 2493	Sun Y, Liu H, Yang B, Wang C, Foo JN, Bao F, Irwanto A, Yu G, Fu X, Wang Z, You J, Liu J, Zhou G, Liu J, Zhang F. Investigation of the predisposing factor of pemphigus and its clinical subtype through a genome-wide association and next generation sequence analysis. J Eur Acad Dermatol Venereol. 2019 Feb;33(2):410-415.
HLA-DQB1	DQB1*05:03	31.16	8.6x10-68	ni							
HLA-DRB1	DRB1*04:06, 14:04, 14:05	2.52, 6.27, 15.86	1.2x10-4, 1.2x10-6, 7.6x10-21	DRB1*09:01, 15:01, 16:02	0.41, 0.27, 0.25	2.9x10-5, 8.5x10-6, 6.5x10-6	95% CI not informed. HLA genotypes were imputed from GWAS data. GWAS results were replicated in a 2nd sample of 110 patients and 604 controls. Groups DRB1*14; DQB1*05 were significantly increased and groups DRB1*08, 09, 15, 16; DQA1*05; DQB1*06 were significantly decreased among patients. After conditional analysis, authors concluded thar only associations with DQB1*05:03 and DQA1*01:01 were independent.	GWAS (microarray)	Chinese (Han)	P: 365; C: 501	Zhang SY, Zhou XY, Zhou XL, Zhang Y, Deng Y, Liao F, Yang M, Xia XY, Zhou YH, Yin DD, Ojaswi P, Hou QQ, Wang L, Zhang DY, Xia DM, Deng YQ, Ding L, Liu HJ, Yan W, Li MM, Ma WT, Ma JJ, Yu Q, Liu B, Yang L, Zhang W, Shu Y, Xu H, Li W. Subtype-specific inherited predisposition to pemphigus in the Chinese population. Br J Dermatol. 2019 Apr;180(4):828-35.
HLA-DQA1	DQA1*01:01	9.11	1.2x10-37	DQA1*01:02, 05:01	0.30, 0.32	7.9x10-12, 4.7x10-7					
HLA-DQB1	DQB1*05:03	16.8	7.3x10-43	DQB1*03:03	0.41	2.6x10-5					

& Association with DRB1*14:06-DQA1*05:03-DQB1*03:01 in Japanese was suggested by Miyagawa et al. 1997

Allele DRB1*14:54 has been missclassified as DRB1*14:01 in most studies exept in Eastern Asian populations (see the main text for the explanation)

P: patients; C: controls; na: not analyzed; ni: not informed;

PCR-SSO: polymerase chain reaction + sequence-specific oligonucleotides; PCR-SSP: PCR with sequence-specific primers; PCR-RFLP: PCR + restriction fragment length polymorphism; SBT: sequence-based typing; GWAS: genome-wide association study; NGS: next-generation sequencing
