# Supplementary material for: Beyond the HLA polymorphism: a complex pattern of genetic susceptibility to pemphigus
Source: Genet Mol Biol. 2020 Jul 1;43(3):e20190369. doi: 10.1590/1678-4685-GMB-2019-0369 (PMC7341728; doi:10.1590/1678-4685-GMB-2019-0369)
Supplement: Supplementary file 2 [file 1415-4757-GMB-43-3-e20190369-s2.pdf]

Supplementary Material to "Beyond the HLA polymorphism: a complex pattern of genetic susceptibility to pemphigus"

Table S2 - HLA class II associations described for pemphigus foliaceus in different populations

Gene	Susceptibility	OR or RR	P value	Protective	OR or RR	P value	Comment	Method	Population	Sample	Reference
HLA-DR individuals with the antigen (allele group)	DR1(DRB1*01), DR4(DRB1*04)	6.4, 3.3	0.00007, 0.003	DR7(DRB1*07)	0.06	0.00019	95% CI not informed. In parenthesis: actual nomenclature of DR and DQ allele groups	microlymphocytotoxicity	Brazilian (admixed of predominantly European Ancestry)	PF(FS): 48, C: 74 (matched)	Petzl-Erler ML, Santamaria J. Are HLA class II genes controlling susceptibility and resistance to Brazilian pemphigus foliaceus (fogo selvagem)? Tissue Antigens. 1989 Mar;33(3):408-14.
HLA-DQ individuals with the antigen (allele group)	DQ1(DQB1*05:01)	3.2	0.018	DQ2(DQB1*02)	0.27	0.0081					
HLA-DR-DQ haplotypes	DR1-DQ1(01-05:01), DR4-DQ3(04-03:02)	na	na	DR7-DQ2(07-01), DR3-DQ2(03-02)	na	na					
HLA-DRB1 individuals with the allele	DRB1*01, 01:01, 01:02, 01:03, 04, 04:04, 04:06, 04:10, 14:06, 16:01	7.40(4.77-11.47), 1.83(1.08-3.12), 10.36(5.92-18.13), 5.41(1.27-22.95), 2.66(1.72-4.11), 4.58(2.16-9.72), 35.85(nd), 9.62(0.99-93.35), 4.04(1.07-15.30), 2.87(1.29-6.37)	<10-6, 0.042; <10-6; 0.025; <10-6, 0.0004 0.0005 0.046, 0.044, 0.017	DRB1*03:01, 07:01, 08, 08:01, 11, 11:01, 11:04, 14:02, 15	0.23(0.09-0.59), 0.09(0.03-0.30), 0.27(0.11-0.64), 0.07(nd), 0.09(0.03-0.28), 0.05(0.01-0.40), 0.15(0.02-1.13), 0.09(nd), 0.51(0.30-0.89)	0.0009, <10-6, 0.0011, 0.007, <10-6, <10-6, 0.037, 0.018, 0.019	Interactions between susceptibility (SU), protective (PR) and neutral (NE) alleles: Protection is dominant, since the PR/NE and PR/PR genotypes are both equally (P=0.95) and strongly protective (OR=0.07 and 0.05, respectively; P<10-6 for both). Risk of SU/SU genotype is higher (P=0.012) than for SU/NE (OR=8.7 and 4.0; P<10-6), For the SU/PR genotype the OR does not differ statistically from 1, consistent with lack of dominance between PR and SU alleles.	PCR-SSO	Brazilian (admixed of predominantly European Ancestry)	PF(FS): 128, C: 402 (matched)	Pavoni DP, Roxo VM, Marquart Filho A, Petzl-Erler ML. Dissecting the associations of endemic pemphigus foliaceus (Fogo Selvagem) with HLA-DRB1 alleles and genotypes. Genes Immun. 2003 Mar;4(2):110-6.
HLA-DQA1 individuals with the allele	DQA1*01:01, 01:03	4.72(2.78-8.03)	<10-5	DQA1*01:03, 02:01, 05:01	0.14 (0.03-0.61), 0.07(0.02-0.28), 0.31(0.17-0.58)	0.002, <10-5, <10-5		PCR-SSO	Brazilian (admixed of predominantly European Ancestry)	PF(FS): 104; C: 162 (matched)	Petzl-Erler and Roxo, unpubluished data
HLA-DQB1 individuals with the allele	DQB1*05, 05:01	6.63(3.72-11.83), 5.61(3.21-9.83)	<10-5, <10-5	DQB1*02, 03:01, 06	0.12(0.05-0.29), 0.46(0.26-0.84, 0.54(0.31-0.96)	<10-5, 0.012, 0.0034					
HLA-DRB1-DQA1-DQB1 haplotypes	01:02-01:01-05:01, 04:04-03-03:02, 04:06-03:01-03:02, 14:06-05:01-03:01, 16:01-01:02-05:02	na	na	03:01-05:01-02:01, 07:01-02:01-02, 08:01-04:01-04:02, 11:01or11:04-05:01-03:01, 14:02-05:01-03:01, 15-01-06	na	na					
HLA-DRB1	DRB1*01:01, 01:02, 04	2.83(1.88-4.28), 6.06(4.32-8.49), 12.70(8.16-19.76)	0.0001, 5x10-10, <0.0001	DRB1*07:01, 11:01, 13:01	0.30(0.13-0.68), 0.07(0.01-0.50), 0.20(0.06-0.62)	0.024, 0.027, 0.027	Susceptibility: group DRB1*01, protective groups: DRB1*07, 11 and 13	PCR-SSP	Brazilian (admixed of predominantly European Ancestry)	PF: 86, C: 1592 (BMT donors)	Brochado MJF, Nascimento DF, Deghaide NHS, Donadi EA, Roselino AM. Data on HLA class I/II profile in Brazilian pemphigus

Gene	Susceptibility	OR or RR	P value	Protective	OR or RR	P value	Comment	Method	Population	Sample	Reference
HLA-DQA1	DQA1*01, 01:02, 03	0.03(0.01-0.08), 1.41(1.16-1.69), 1.77(1.23-2.54)	0.02, 0.01, 0.0019	DQA1*02:01, 05	0.34(0.15-0.75), 0.30(0.18-0.49)	0.03, <0.0001					patients. Data Brief. 2016 Jun 3;8:364-74.
HLA-DQB1	DQB1*05:01	2.95	3x10-10	DQB1*03:01, 06:03	0.39, 0.22	0.0002, 0.02	95% CI not informed.				
HLA-DRB1-DQA1-DQB1 haplotypes	04-03-04, 01-01-05, 16-01-05	6.2, 4,2, 2,8	0.0001, 0.0001, 0.0023	11-05-03, 13-01-06, 07-02-02	0.13, 0.15, 0.29	6x10-8, 7x10-7, 0.0055	95% CI not informed.	PCR-SSP	Brazilian (admixed of predominantly European Ancestry)	PF: 86, C: 1592 (BMT donors)	Brochado MJF, Nascimento DF, Deghaide NHS, Donadi EA, Roselino AM. Data on HLA class I/II profile in Brazilian pemphigus patients. Data Brief. 2016 Jun 3;8:364-74.
HLA-DRB1 individuals with the allele	DRB1:01(01:01+01:02), 16:01	4.63(1.74-12.34), 8.57(0.98-75.18)	0.0017, 0.0418	DRB1*15	0.19(0.04-0.96)	0.054	Frequencies of the non-associated DRB1, DQA1 and DQB1 alleles and allele groups were not informed. OR and P calculated on basis of the data presented in the manuscript. The p-values for DRB1*15 and 16:01 are borderline. DRB1*04:04 is non-significantly increased among patients (OR=2.22, p=0.23)	PCR-SSO	Brazilian (admixed)	PF(FS): 34; C: 41 (matched)	Moraes ME, Fernandez-Vina M, Lazaro A, Diaz LA, Filho GH, Friedman H, Rivitti E, Aoki V, Stastny P, Moraes JR. An epitope in the third hypervariable region of the DRB1 gene is involved in the susceptibility to endemic pemphigus foliaceus (fogo selvagem) in three different Brazilian populations. Tissue Antigens. 1997 Jan;49(1):35-40.
HLA-DRB1 individuals with the allele	DRB1*04:04	9.60(2.30-40.11)	0.0026	DRB1*04:07, 08:02	na, 0.13(0.02-1.08)	0.0345, 0.0403	OR and P for DRB1*08:02 and 04:07 calculated on basis of the data presented in the manuscript. DRB1*14:02 was not significantlly increased among patients (OR=3.79, p=0.104; haplotype 14:02-05:01-03:01). Neutral allele: 16:02 (OR≅1, p=0.74)	PCR-SSO	Xavante Amerindians	PF(FS): 10; C: 74 (matched)	Cerna M, Fernandez-Viña M, Friedman H, Moraes JR, Moraes ME, Diaz L, Stastny P. Genetic markers for susceptibility to endemic Brazilian pemphigus foliaceus (Fogo Selvagem) in Xavante Indians. Tissue Antigens. 1993 Sep;42(3):138-40.
HLA-DRB1-DQA1-DQB1 haplotypes	04:04-03-03:02	na	na	08:02-04:01-04:02	na	na					
HLA-DRB1 individuals with the allele	DRB1*04:04	6.12(2.07-18.02)	0.0014	none			OR and P calculated on basis of the data presented in the manuscript. Haplotype 14:02-03:01 was non-significantly increased among patients (OR=2.72,p=0.079). Haplotype 08:02-04:02 was non-significantly decreased among patients (OR=0.24, p=0.082). Neutral DRB1-DQB1 haplotypes (OR≅1, p>0.57) 04:07-03:02, 04:11-03:02, 14:06-03:01, 16:02-03:01	PCR-SSO	Terena Amerindians	PF(FS): 20; C: 66 (matched)	Moraes ME, Fernandez-Vina M, Lazaro A, Diaz LA, Filho GH, Friedman H, Rivitti E, Aoki V, Stastny P, Moraes JR. An epitope in the third hypervariable region of the DRB1 gene is involved in the susceptibility to endemic pemphigus foliaceus (fogo selvagem) in three different Brazilian populations. Tissue Antigens. 1997 Jan;49(1):35-40.
HLA-DRB1-DQB1 haplotypes	04:04-03:02	na	na								
HLA-DRB1 allele frequencies	DRB1*04:02, 13:02, 14:01	7.0, 13.6, 21.64	0.038, 0.0015, 0.009	[DRB1*11 absent in patients]		ns	95% CI not informed. DRB1*01: 8,8% in P, 9.5% in C, no high resolution; DRB1*07 8.8% in P, 8.4% in C, 08: 2.9% in P, 6.5% in C, 11: 0% in P, 10.4% in C	PCR-SSO	Venezuelan (admixed)	PF: 17; C: 101	Sáenz-Cantele AM, Fernández-Mestre M, Montagnani S, Calebotta A, Balbas O, Layrisse Z. HLA-DRB1*0402 haplotypes without DQB1*0302 in Venezuelan patients with pemphigus vulgaris. Tissue Antigens. 2007 Apr;69(4):318-25.
HLA-DQB1 allele frequencies	DQB1*05:03	3.32	0.05	DQB1*03:01	0.16	0.007	95% CI not informed. DQB1*02: 11.7% in P, 14.4% in C				
HLA-DRB1 individuals with the allele	DRB1*03, 04	3.25, 3.00	0.00048, 0.0007	DRB1*11, 15	0.07, 0.09	0.01, 0.04	95% CI not informed. DRB1*13 was non-significantly decreased among patients (OR=0.33, p=0.09). Only for groups DRB1*03 and 04 high resolution typing was informed.	PCR-SSP	Tunisian (south)	PF(endemic): 50; C: 130	Abida O, Zitouni M, Kallel-Sellami M, Mahfoudh N, Kammoun A, Ben Ayed M, Masmoudi A, Mokni M, Fezzaa B, Ben Osman A, Kammoun MR, Turki H, Makni H, Gilbert D, Joly

Gene	Susceptibility	OR or RR	P value	Protective	OR or RR	P value	Comment	Method	Population	Sample	Reference
HLA-DQB1 individuals with the allele	DQB1*03:02	2.76	0.004	DQB1*03:01, 06	0.21, 0.30	0.007, 0.013					P, Tron F, Makni S, Masmoudi H; Franco-Tunisian Group for Survey and Research on Pemphigus. Tunisian endemic pemphigus foliaceus is associated with the HLA-DR3 gene: anti-desmoglein 1 antibody-positive healthy subjects bear protective alleles. Br J Dermatol. 2009 Sep;161(3):522-7.
HLA-DRB1 individuals with the allele	DRB1*04, 04:02, 04:04	2.44, ni, ni	0.02, 0.01, 0.03	DRB1*04:05	ni	0.0002	95% CI not informed. DRB1*11 was non-significantly decreased among patients (OR=0.23, p=0.11). Only for groups DRB1*03 and 04 high resolution typing was informed.	PCR-SSP	Tunisian (north)	PF(sporadic): 40; C: 140	Abida O, Zitouni M, Kallel-Sellami M, Mahfoudh N, Kammoun A, Ben Ayed M, Masmoudi A, Mokni M, Fezzaa B, Ben Osman A, Kammoun MR, Turki H, Makni H, Gilbert D, Joly P, Tron F, Makni S, Masmoudi H; Franco-Tunisian Group for Survey and Research on Pemphigus. Tunisian endemic pemphigus foliaceus is associated with the HLA-DR3 gene: anti-desmoglein 1 antibody-positive healthy subjects bear protective alleles. Br J Dermatol. 2009 Sep;161(3):522-7.
HLA-DQB1 individuals with the allele				DQB1*03:01	0.17	0.007					
HLA-DRB1	DRB1*01:02, 04, 04:02, 04:06, 14, 14:04	3.84, 2.71, 8.89, 20.75, 2.90, 27.65	0.04, 0.01, 0.02, 0.003, 0.04, 0.0008				95% CI not informed. DRB1*03 and 07 non-significantly decreased among patients (OR=0.25 p=0.094; OR=0.48 p=0.229). Only for groups DRB1*01, 04 and 14 high resolution (4-digit) typing was informed.	PCR-SSO for low resolution, plus PCR-SSP and PCR-RFLP for 4-digit typings	French	PF(sporadic): 31, C: 84	Martel P, Gilbert D, Busson M, Loiseau P, Lepage V, Drouot L, Delaporte E, Prost C, Joly P, Charron D, Tron F. Epistasis between DSG1 and HLA class II genes in pemphigus foliaceus. Genes Immun. 2002 Jun;3(4):205-10.
HLA-DQB1	DQB1*03:02	3.00	0.02	DQB1*02(02:01+02:02)	0.32	0.02	DQB1*05:03 non-significantly increased among patients (OR=2.55, p=0.08).			PF(sporadic): 30, C: 64	
HLA-DRB1	DRB1*04:06	5.58	7.4x10-13	ni			HLA genotypes were imputed from GWAS data and validated by NGS. 95% CI not informed.	GWAS (microarray) and NGS-based HLA typing	Chinese (Han)	PF(sporadic): 159; C: 2493	Sun Y, Liu H, Yang B, Wang C, Foo JN, Bao F, Irwanto A, Yu G, Fu X, Wang Z, You J, Liu J, Zhou G, Liu J, Zhang F. Investigation of the predisposing factor of pemphigus and its clinical subtype through a genome-wide association and next generation sequence analysis. J Eur Acad Dermatol Venereol. 2019 Feb;33(2):410-415.
HLA-DQB1	DQB1*05:03	5.64	4.8x10-17	ni							
HLA-DRB1	DRB1*04:06, 14:05	4.28, 6.15	1.6x10-5, 1.6x10-5	none			GWAS results for HLA were are replicated in a 2nd sample of 32 patients and 604 controls. Groups DRB1*04 and 14 were significantly increased among patients. After conditional analysis, authors concluded that only associations with DQB1*05:03 and DQA1*01:01 were independent. 95% CI not informed.	GWAS (microarray)	Chinese (Han)	PF(sporadic): 72; C: 501	Zhang SY, Zhou XY, Zhou XL, Zhang Y, Deng Y, Liao F, Yang M, Xia XY, Zhou YH, Yin DD, Ojaswi P, Hou QQ, Wang L, Zhang DY, Xia DM, Deng YQ, Ding L, Liu HJ, Yan W, Li MM, Ma WT, Ma JJ, Yu Q, Liu B, Yang L, Zhang W, Shu Y, Xu H, Li W. Subtype-specific inherited predisposition to pemphigus in the Chinese population. Br J Dermatol. 2019 Apr;180(4):828-35.
HLA-DQA1	none			DQA1*01:02	0.34	2x10-4					
HLA-DQB1	DQB1*03:02, 05:03	3.35, 4.63	7.2x10-6, 2.4x10-8	none							

In red: unique associations, not reported in other populations or studies.

P: patients; C: controls; na: not analyzed; ni: not informed;

PCR-SSO: polymerase chain reaction + sequence-specific oligonucleotides; PCR-SSP: PCR with sequence-specific primers; PCR-RFLP: PCR + restriction fragment length polymorphism; GWAS: genome-wide association study; NGS: next-generation sequencing
